# Evidence for a Role of the Transcriptional Regulator Maid in Tumorigenesis and Aging

**DOI:** 10.1371/journal.pone.0129950

**Published:** 2015-06-24

**Authors:** Koichi Fujisawa, Shuji Terai, Toshihiko Matsumoto, Taro Takami, Naoki Yamamoto, Hiroshi Nishina, Makoto Furutani-Seiki, Isao Sakaida

**Affiliations:** 1 Center for Reparative Medicine, Yamaguchi University School of Medicine, Minami Kogushi 1-1-1, Ube Yamaguchi 755–8505, Japan; 2 Department of Gastroenterology and Hepatology, Yamaguchi University Graduate School of Medicine, Minami Kogushi 1-1-1, Ube Yamaguchi 755–8505, Japan; 3 Division of Gastroenterology and Hepatology, Graduate School of Medical and Dental Sciences, Niigata University, 1–757 Asahimachidori, Chuo-Ku, Niigata 951–8510, Japan; 4 Department of Developmental and Regenerative Biology, Medical Research Institute, Tokyo Medical and Dental University, 1-5-45 Yushima, Bunkyo-ku, Tokyo 113–8510, Japan; 5 Department of Biology and Biochemistry, University of Bath, Bath BA2 7AY, United Kingdom; University of Udine, ITALY

## Abstract

Maid is a helix-loop-helix protein that is involved in cell proliferation. In order to further elucidate its physiological functions, we studied Maid activity in two small fish model systems. We found that Maid expression was greatest in zebrafish liver and that it increased following partial hepatectomy. Maid levels were also high in hepatic preneoplastic foci induced by treatment of zebrafish with diethylnitrosamine (DEN), but low in hepatocellular carcinomas (HCC), mixed tumors, and cholangiocarcinomas developing in these animals. In DEN-treated transgenic medaka overexpressing Maid, hepatic BrdU uptake and proliferation were reduced. After successive breedings, Maid transgenic medaka exhibited decreased movement and a higher incidence of abnormal spine curvature, possibly due to the senescence of spinal cord cells. Taken together, our results suggest that Maid levels can influence the progression of liver cancer. In conclusion, we found that Maid is important regulator of hepatocarconogenesis and aging.

## Introduction

Maid was first isolated from mouse embryos and showed that it has Helix-loop-helix (HLH) structure [1]. Maid is also known as CCNDBP1 (Cyclin D1 binding protein), GCIP (Grap2 and cyclin D-interacting protein) and DIP1 (D type cyclin interacting protein 1), and plays a putative role in the inhibition of E2F-1 transcription factor activity [2–4]. We previously demonstrated that Maid is a useful marker for human hepatocarcinogenesis since its expression is high in hepatic preneoplastic foci and decreases as cells become progressively less differentiated [5]. Ma et al. examined transgenic mice overexpressing Maid and found that there were no obvious changes in liver development or liver regeneration; however, these mice were less susceptible than wild type (WT) animals to chemical hepatocarcinogenesis induced by diethylnitrosamine (DEN) [6]. Sonnenberg et al. generated Maid knockout (KO) mice, and observed that Maid is a tumor suppressor gene because hepatocellular carcinoma (HCC) development was observed [7]. Consistent with this observation, Chen et al. found that Maid expression was lowest in human breast cancers with very poor prognoses [8]. Other work in human cancer cells has indicated that “Ras associated with diabetes” (Rad) can interact with Maid and block its tumor suppressor activity. Furthermore, when Rad was knocked down in a human cancer cell line, Maid translocated to the nucleus and induced cell cycle arrest and senescence [9]. These results suggest that Maid may also be involved in aging.

Despite the above body of work, it has been difficult to dissect the mechanisms of Maid functions in mammals. To gain deeper insight into Maid mechanisms, we examined its biology in two small fish models. Small fish like zebrafish (*Danio rerio*) and medaka (*Oryzias latipes*) can therefore be used to model many aspects of tumorigenesis [10]. Indeed, prior research has demonstrated that a variety of cancers, including hepatic malignancies, can be induced by carcinogens in both these species [11, 12]. Thus, to investigate in more depth the functions of Maid in cancer and aging, we characterized Maid expression and functions in zebrafish and transgenic medaka expressing Human Homolog of Maid (HHM). Here, we report the results of our initial investigations of Maid biology in these model systems.

## Materials and Methods

### Zebrafish and medaka strains

The RIKEN WT (RW) zebrafish strain (*Danio rerio)* was obtained from the Riken stock center (the support of National Bioresource Project of Japan) and maintained at 28°C with a 14 h light/10 h dark cycle. Zebrafish were fed living brine shrimp and flake food (Kyowa N type N700). The inbred medaka strain (Kyoto-Cab) was used in this study (Furutani-Seiki et al., 2004). The protocol was approved by the Committee on the Ethics of Animal Experiments of the University of Yamaguchi university (Permit Number: 21S08). All surgery for rabbits or zebrafish was performed under sodium pentobarbital anesthesia or tricaine anesthesia, respectively. All efforts were made to minimize suffering during the course of this study. Rabbit and fish were sacrificed by overdose of anesthesia.

### Western blot analysis

Protein lysates were obtained by homogenizing tissues or cell pellets in sample buffer containing 62.5 mM Tris–HCl (pH 6.8), 4% SDS, 200 mM dithiothreitol, 10% glycerol, and 0.001% bromophenol blue at a ratio of 1:10 (w/v), followed by boiling. Western blot analysis was performed on 15% SDS-PAGE as described [13].

### Antibodies

To obtain polyclonal anti-Maid antibody, a plasmid containing genes for His-tagged zebrafish Maid was prepared and expressed in *E*. *coli* using standard protocols. The resulting Maid protein was purified using an anti-His antibody affinity column. Purified Maid protein was injected into the skin of rabbits to raise anti-Maid antibodies according to standard protocols. Polyclonal anti-HHM antibody was raised in rabbits as previously [14].

### Cell lines

Zebrafish cell lines (ZF-L, CRL-2643) were procured from ATCC. The cells were cultured in accordance with a slightly modified version of the protocol recommended by ATCC.

### Isolation of zebrafish Maid cDNA

A BLAST search was carried out using the human (BC008188) and mouse (BC002187) Maid protein sequences and the *Danio rerio* BLAST Server (http://www.sanger.ac.uk/cgi-bin/blast/submitblast/d_rerio). The genomic sequence of *Danio rerio* Maid was identified in Ensembl (http://www.ensembl.org/Danio_rerio/index.html). Based on these analyses, the entire coding region of Maid was PCR-amplified and sequenced using the primers: 5' TGCAGACACGCACAGGTTAT-3' (forward) and 5'-AGCGGTGCGATTGTAAATG-3' (reverse).

### Sequence alignment and phylogenetic tree

The amino acid sequences of Maid in 7 species were obtained from the National Center for Biotechnology Information databases and aligned using ClustalW (http://www.ddbj.nig.ac.jp/). A phylogenetic tree created from ClustalW alignment data was generated using TreeView (http://taxonomy.zoology.gla.ac.uk/rod/treeview.html).

### Real-time quantitative PCR

Total RNA was isolated using an RNeasy-kit (Qiagen GmbH, Hilden, Germany) according to the manufacturer's instructions. For cDNA synthesis, Taqman reverse transcription reagents (Roche Diagnostics, Indianapolis, IN, USA) were used as described in the manufacturer's manual. Relative quantification of gene expression was also performed as described in the manual, using Rpl13a and Ef1a as internal controls. The threshold cycle and the standard curve method were used to calculate the relative amount of the target RNA. Light Cycler Q-PCR (Roche Diagnostics) was performed (operating system version 3.0) in 13 μL reaction mixtures containing 2 μL Faststart DNA Master SYBR Green I, 25 μM MgCl_2_, 10 μM of each primer, and 5 μg of extracted DNA. The reaction was performed with preliminary denaturation for 10 min at 95°C (slope 20°C/s), followed by 40 cycles of denaturation at 95°C for 15 s (slope 20°C/s), annealing at 68°C for 5 s (slope, 20°C/s), primer extension at 72°C for 10 s (slope 20°C/s), and product detection at 72°C for 60 s (slope 20°C/s). The synthesis kit for RT-PCR (AMV) and Light Cycler-Faststart DNA Master SYBR Green I were purchased from Roche Diagnostics. Advantage PCR polymerase was obtained from Clonetech Laboratories Inc. (Palo Alto, CA). Primer sequences were as follows:

Cyclin D1 Forward: 5'-GCTCGAGGTCTGTGAAGAGC-3'

Reverse: 5'-GGTGGGCTCCACAGATAAAA-3'

Rpl 13a Forward: 5'-TCTGGAGGACTGTAAGAGGTATGC-3'

Reverse: 5'-AGACGCACAATCTTGAGAGCAG-3'

EF1a Forward: 5'-CTGGAGGCCAGCTCAAACAT-3'

Reverse: 5'-ATCAAGAAGAGTAGTACCGCTAGCATTAC-3'

Maid Forward: 5'-AGTCCAGGAGTCACACTGAG-3'

Reverse: 5'-GGTGAGGACAGAATGACATC-3'

### Gene expression microarrays

The cRNA was amplified, labeled, and hybridized to a oligomicroarray according to the manufacturer's instructions. All hybridized microarray slides were scanned by an Agilent scanner. Relative hybridization intensities and background hybridization values were calculated using Agilent Feature Extraction Software (9.5.1.1). The raw signal intensities of all samples were log2-transformed and normalized. We selected the probes, excluding the control probes, where the detection p-values of all samples were less than 0.01 and use them to identify differentially expressed genes.

### Immunohistochemistry

Tissue samples prepared from zebrafish were fixed in 4% paraformaldehyde and sliced into 3 μm sections using a cryostat. Prior to immunohistochemical staining, endogenous peroxidase in fixed tissue slices was blocked by treatment with fresh 0.3% hydrogen peroxidase in methanol for 30 min at 4°C. Blocked samples were incubated with polyclonal rabbit anti-zebrafish Maid and rabbit anti-HHM antibodies (1:5000) overnight at 4°C according to an established protocol [14]. After washing 3 times in PBS, the sections were incubated with biotin-conjugated secondary antibody in PBS for 3 h at 20°C. After 3 additional PBS washes, a peroxidase–anti-peroxidase complex and streptavidin were added and incubation was maintained at 20°C. Positive reactions were developed for 5–10 min using Tris–HCl buffer containing hydrogen peroxidase and 3,3′-diaminobenzidine. Normal goat serum (Vector Laboratories, Burlingame, CA, USA) was used as a negative control.

### Morpholino knock-down experiments

Maid anti-sense morpholino (5'-CAT GAC GCG CTT TGC TTC AGT CTC G-3'), as well as the standard control morpholino (5'-CCT CTT ACC TCA GTT ACA ATT TAT A-3'), were purchased from Gene Tool (USA). Morpholinos (10 μM final concentration) were microinjected into one-cell stage zebrafish embryos.

### Partial hepatectomy and fin amputation

Partial hepatectomy was performed as previously described [15]. Briefly, fish were anesthetized, the abdomen was resected, and part of the ventrical lobe of the liver was removed using a scalpel. Liver regeneration was monitored for 7 days. For fin amputation, fins were resected with a scalpel at 1/3 of the total distance from the tip. Fin regeneration was monitored for 14 days.

### Chemical carcinogenesis

For zebrafish studies, one-year-old zebrafish were maintained in 6 L of water containing 200 ppm diethylnitrosamine (DEN; Sigma Chemical Co, St Louis MO.) for 3 or 8 weeks, with the water changed once per week. Treated fish were then exposed to 75 mg/L 5-bromo-2′-deoxyuridine (BrdU) solution (Sigma Chemical Co, St Louis MO.) for two days. Fish were sacrificed and cell proliferation analyzed by immunohistochemistry. Preneoplastic foci and tumor types were identified by their size and pattern of HE staining under a light microscope. For HHM transgenic medaka studies (see below), fish were reared in 6 L of water containing 100 ppm DEN for 3 weeks, during which the water was changed weekly. Treated fish were then exposed to 75 mg/L BrdU for 2 days as described above [16]. Fish were sacrificed and cell proliferation analyzed as above. Homozygous, heterozygous and control HHM transgenic medaka were all reared in the same aquarium during DEN treatment.

### Generation of HHM transgenic medaka

For HHM expression, a pcDNA3.1 vector was constructed that contained: (1) the HHM coding domain placed under the control of the promoter of the red sea bream β-actin gene, and (2) GFP under the control of a separate red sea bream β-actin promoter.

To establish transgenic medaka strains that ubiquitously expressed Maid, 10 ng/μl circular DNA containing the HHM cDNA under the control of the red sea bream β-actin promoter was injected into one-cell stage embryos of the Kyoto-Cab inbred strain. The circular DNA was prepared using a Qiagen EndoFree plasmid Maxiprep kit (Qiagen, USA). EGFP-positive embryos were selected by fluorescence microscopy, allowed to mature, and bred to WT fish of the Kyoto-Cab inbred strain at 28°C. To identify HHM transgenic fish, livers were isolated from GFP-positive individuals and HHM expression was determined by Western blotting. Representative lines (31 and 52) were selected for experiments. Fish heterozygous for the HHM transgene and non-transgenic siblings were maintained as controls. Transgenic medaka and siblings born on the same day were reared in the same aquarium and used in the same experiment. For chemical carcinogenesis, HHM transgenic medaka were treated with DEN as described [14]. Evaluation of movement was performed with the chronobiology kit for fish.

### SA-β-gal staining

Tissue slices (1 mm) were cut from the area of the anus in medaka and stained for 12 hrs using a SA-β-gal staining kit (Biovision) for visualization of senescent cells.

### Statistical analyses

All data are expressed as the mean ± S.D. unless otherwise indicated. One way ANOVA followed by the Dunnett post hoc multiple comparison test was performed to assess the statistical significance of differences in the RT-PCR analysis of *Maid* mRNA expression after PH and fin amputation, and the quantitation of the BrdU labeling results. The Student’s t-test was performed to assess the results of the real-time RT-PCR analysis and to compare the body lengths of transgenic medaka. P values less than 0.05 were considered to be significant. Mann-Whitney U test and Student’s t-test were performed to assess the statistical significance of differences in neoplastic and tumors between group1 and group2.

## Results

### cDNA sequence and amino acid sequence comparison

The full length cDNA contained 1210 bp and encoded a 344 amino acid protein that contained putative leucine zipper and HLH domains (S1 Fig). Maid is present in vertebrates, including fish such as the puffer fish and stickleback (S2 Fig). The homology of ZHM at the amino acid level is 38% to humans, 48% to chickens, 43% to Xenopus, 48% to tetraodontidae and 59% to sticklebacks.

### Maid expression in normal adult zebrafish

**Fig 1:**
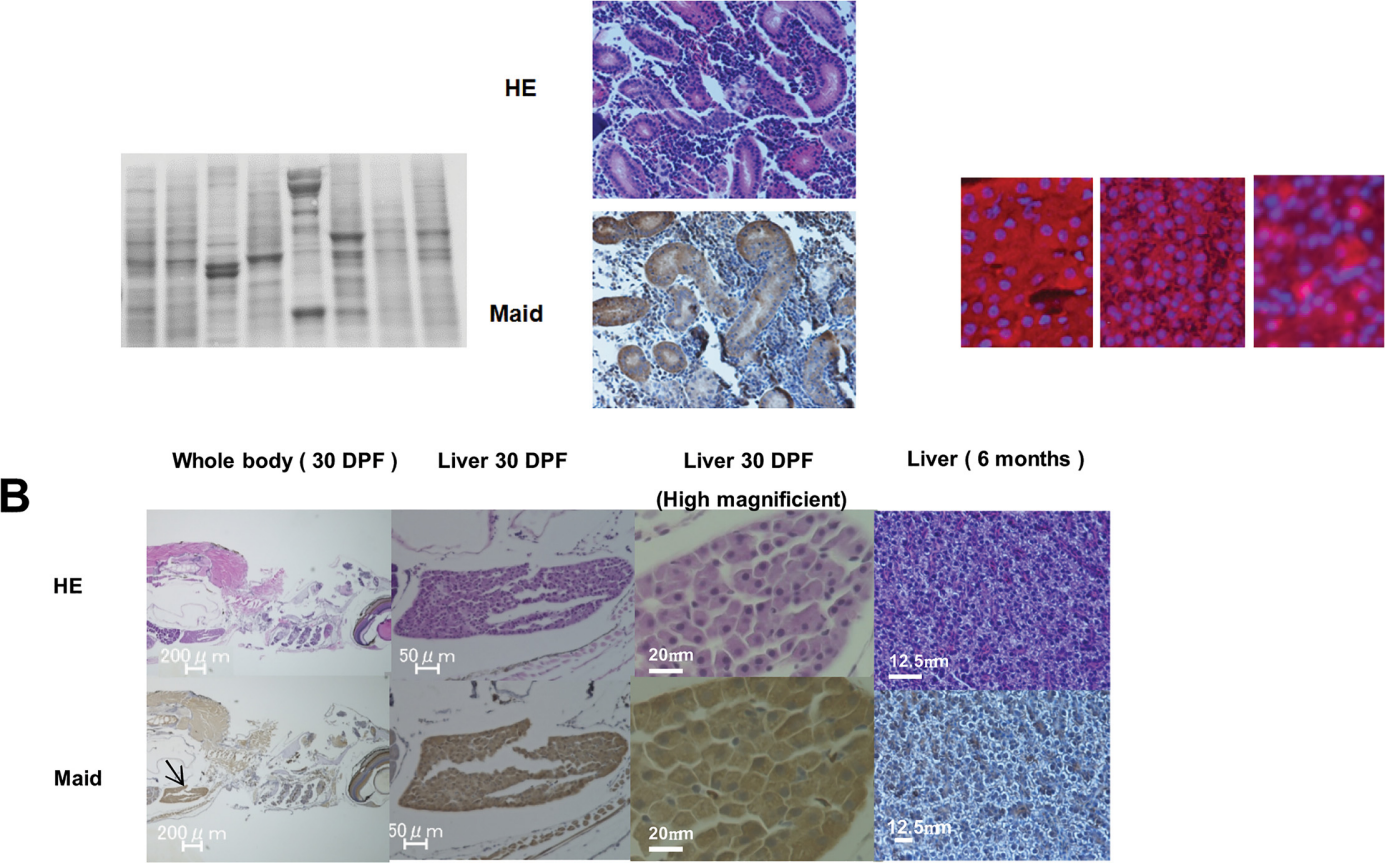
Zebrafish Maid expression in adult tissues. (A) Western blot to detect Maid in total protein isolated from the indicated tissues of adult zebrafish. Ponseau-S staining shows total protein bands. (B) Top: Hematoxylin and eosin (HE) staining of the whole body and liver of a WT zebrafish embryo at 30 dpf, and the liver of a WT adult zebrafish at 6 months of age. Bottom: Immunohistochemical staining to detect Maid in the zebrafish tissues in the top panel. Black arrow indicates the liver, where the highest level of Maid expression was observed. (C) Top: HE staining of zebrafish kidney at 6 months of age. Bottom: Immunohistochemical staining to detect Maid in the zebrafish kidney in the top panel. (D) Localization of Maid in zebrafish liver. Immunofluorescent analysis of Maid protein localization in untreated juvenile zebrafish liver, adult zebrafish liver, and a DEN-induced foci. Red, anti-zebrafish Maid antibody; blue, DAPI (visualization of nuclei).

To initiate our delineation of Maid functions in small fish, we examined levels of Maid protein in various tissues of adult zebrafish using Western blotting and Ponseau-S staining. The liver showed the highest Maid levels, although relatively high expression was also noted in the intestine, kidney, and testis (Fig 1A). Similarly, immunohistochemical staining demonstrated that the highest expression of Maid protein occurred in the liver of zebrafish larvae at 30 days post-fertilization (30 dpf) (Fig 1B), with localization predominantly in the cytoplasm of hepatocytes (Fig 1D left*)*. Compared with larvae, hepatocytes in the livers of adult zebrafish showed lower levels of Maid in both the nucleus and cytoplasm (Fig 1B and 1D middle). Moderate Maid expression was observed in the kidneys and uriniferous tubules (Fig 1C).

**Fig 2:**
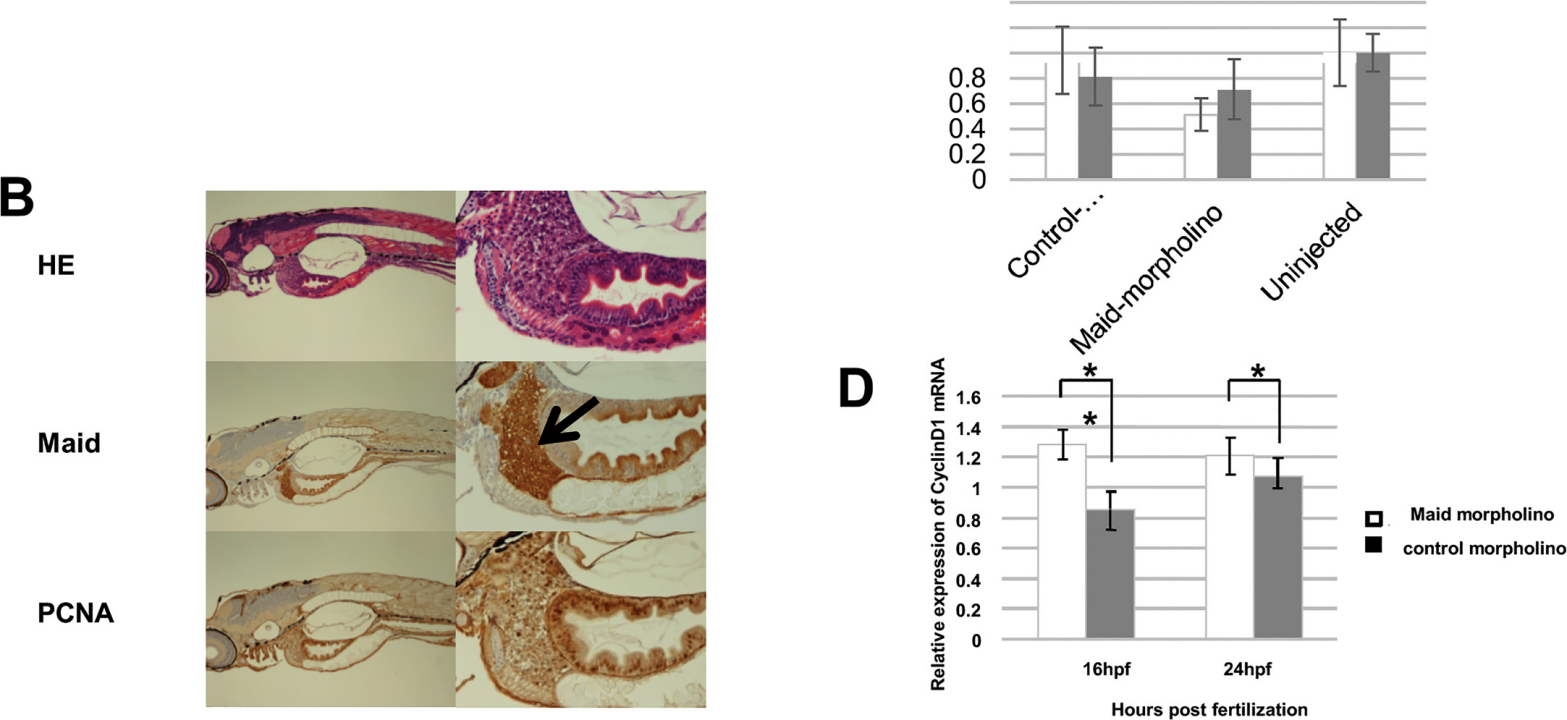
Maid expression during zebrafish development. (A) Western blot analysis of Maid expression: lane 1, ovary; 2, unfertilized egg (0 h); 3, cleavage (1.5 h); 4, blastula (4 h); 5, gastrula (10 h); 6, segmentation (20 h), and 7, hatching (72 h). Ponseau staining as a loading control. Macroscopic views of developing zebrafish embryos are also shown. (B) HE staining of whole zebrafish at the hatching stage shown at low (left) and high (right) magnifications. Black arrow indicates the liver. (C) Top: Fertilized zebrafish eggs were left uninjected (control, lane 3), or microinjected at the one- to two-cell stage with control morpholino (lane 1) or morpholino against Maid (ZHM) (lane 2). Middle: Western blot analysis was performed to detect Maid in protein isolated from these embryos at 16 hpf or 24 hpf. Results are representative of 20 embryos examined per treatment. Bottom: Graphical representation of Western blot analysis, *, p<0.05. (D) Real-time RT-PCR analysis of *cyclin D1* mRNA expression in extracts of the treated zebrafish embryos in (C). Results are the mean ± SD (n = 20/group) and are expressed relative to levels of *El1a* mRNA (set to 1 as internal control). *, p<0.05; **, p<0.01.

We next analyzed Maid expression during zebrafish embryogenesis. High levels of *Maid* mRNA expression have been documented in human fetal liver [2], and *in situ* hybridization studies have detected Maid protein in the liver, central nervous system, and dorsal root ganglia of fetal mice [7]. No previous report, however, has addressed changes in Maid protein levels during development. We therefore monitored Maid protein expression during zebrafish embryogenesis and found that maternal-derived protein was present before fertilization, and that Maid protein levels increased as embryonic development progressed (Fig 2A). Immunohistochemical staining of larvae immediately after hatching revealed high PCNA and Maid expression in the liver, as expected (Fig 2B). Because these results suggested that Maid might play an important role during embryogenesis, we examined the effect of inhibiting Maid expression during zebrafish development using a specific morpholino antisense oligonucleotide [17]. We confirmed that morpholino injection into one-cell embryos resulted in inhibition of Maid protein production. Nevertheless, the effect of Maid protein knockdown was significant at 16 hours post-fertilization (hpf) and 24 hpf (Fig 2C), no significant changes in larval morphology or time of hatching were observed. Interestingly, analysis of gene expression using real-time PCR revealed that *cyclin D1* mRNA was significantly elevated in Maid morpholino-knockdown embryos at 16 and 24 hpf (Fig 2D).

### Maid expression during tissue regeneration after partial hepatectomy or fin amputation

**Fig 3:**
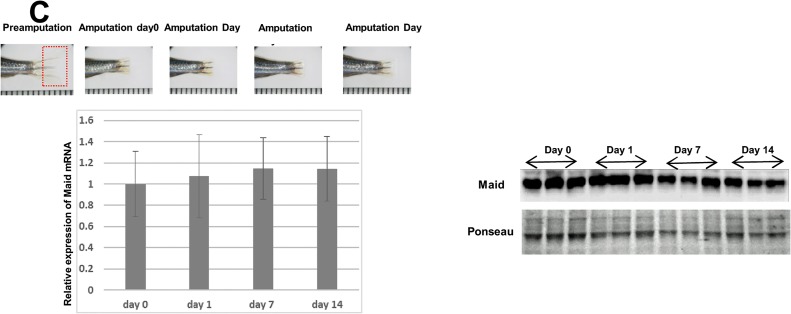
Maid expression following partial hepatectomy or fin amputation in zebrafish. (A) Top: Diagram of the area of adult zebrafish liver subjected to PH. Middle: Macroscopic views of fish abdomens, sliced in the middle. About 30% of the ventral lobe of the liver was removed. The area of the liver regeneration is demarcated by a rectangle. Bottom: Macroscopic view of the livers in the middle panel after 7 days of regeneration. Results are representative of 10 fish per group. (B) Left: RT-PCR analysis of *Maid* mRNA expression in regenerated zebrafish liver at the indicated times after PH. Results are the mean ± SD expressed relative to Gapdh and are representative of at least 5 fish per group, *, p<0.05. Right: Western blotting analysis of Maid in regenerated zebrafish liver. (C) Left Top: Macroscopic views of a representative adult zebrafish tail fin before and after amputation. Fin regeneration was monitored for 14 days. Left Bottom: RT-PCR analysis of *Maid* mRNA expression in regenerating fin at the indicated times after fin amputation. Results are the mean ± SD (n = 3/group) and are expressed relative to *El1a* mRNA in fish at Pre-Amputation. Right: Western blotting analysis of Maid in regenerating fin.

The high levels of Maid expression in zebrafish liver led us to hypothesize that Maid might play a critical role in this organ. We therefore examined Maid expression during liver regeneration in WT zebrafish subjected to PH. We found that the resected livers regenerated to their original size in about 7 days (Fig 3A). Significantly increased amounts of *Maid* mRNA were present in the liver on day 1 after resection, but these had returned to their pre-resection levels by day 7 (Fig 3B). Howerver no statistical changes were not observed in Westernblotting. We also studied Maid expression during fin regeneration, which is the most frequently examined zebrafish tissue regeneration model [15]. However, no significant changes in *Maid* levels in mRNA and protein were observed in the regenerating fin at any time point studied (Fig 3C). These results are consistent with the notion that Maid function is of particular significance for the proliferation of cells during regeneration in the liver but not in the fin.

### Maid expression during DEN-induced liver carcinogenesis

**Fig 4:**
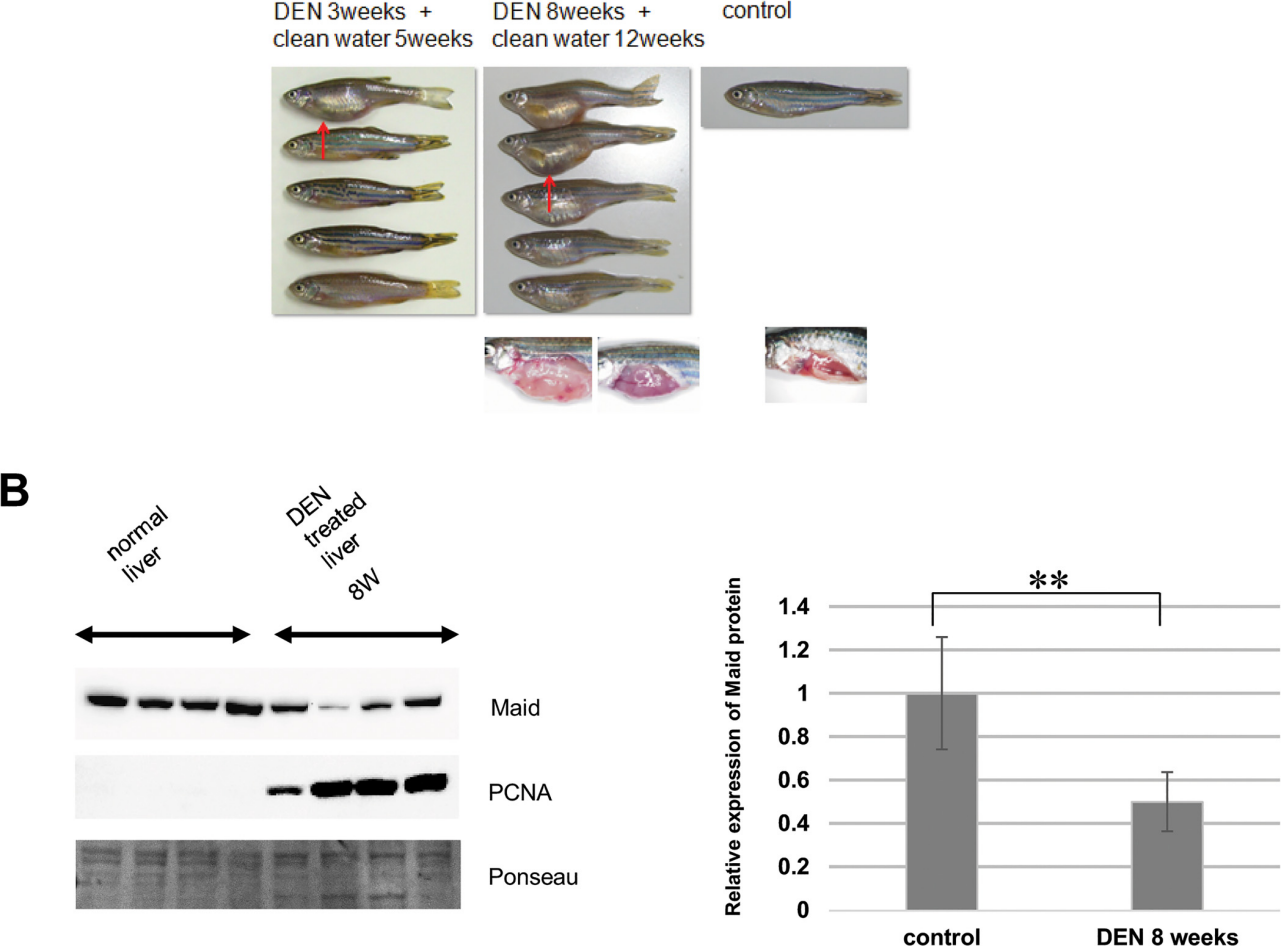
Liver expansion and reduced Maid expression during chemical carcinogenesis in zebrafish. (A) Top left: Macroscopic views of zebrafish in Group 1. Red arrow indicates a distended abdomen. Top middle: Macroscopic views of zebrafish in Group 2. Top right: Macroscopic view of a control zebrafish reared for 5 weeks in clean water and not exposed to DEN. Bottom panels show macroscopic views of (middle) livers of two DEN-treated zebrafish in Group 2 and (right) healthy control liver. (B) Western blot analysis of Maid and PCNA protein in two normal zebrafish livers and livers of 5 zebrafish subjected to 8 weeks of DEN administration. Left: Graphical representation of Western blot analysis, **, p<0.01.

To determine the role of Maid during carcinogenesis in small fish, we treated zebrafish with DEN to induce liver cancer. We studied both a short-term group of fish (n = 46) that were reared in fresh water for 5 weeks after 3 weeks of immersion in water containing 100 ppm DEN (Group 1), and a longer-term group (n = 50) that were reared in fresh water for 12 weeks after 8 weeks of immersion in water containing 100 ppm DEN (Group 2). A comparison of these two groups at their endpoints revealed that Group 2 contained more individuals with distended abdomens (Fig 4A, top). When these fish were autopsied, they had extremely swollen livers (Fig 4A, bottom). Western blotting revealed significant decreased expression of Maid in whole swollen livers from Group 2 fish treated with DEN for 8 weeks compared to normal livers (Fig 4B). In addition, the proliferation marker PCNA was virtually absent from normal livers but elevated in livers from fish treated with DEN for 8 weeks.

**Fig 5:**
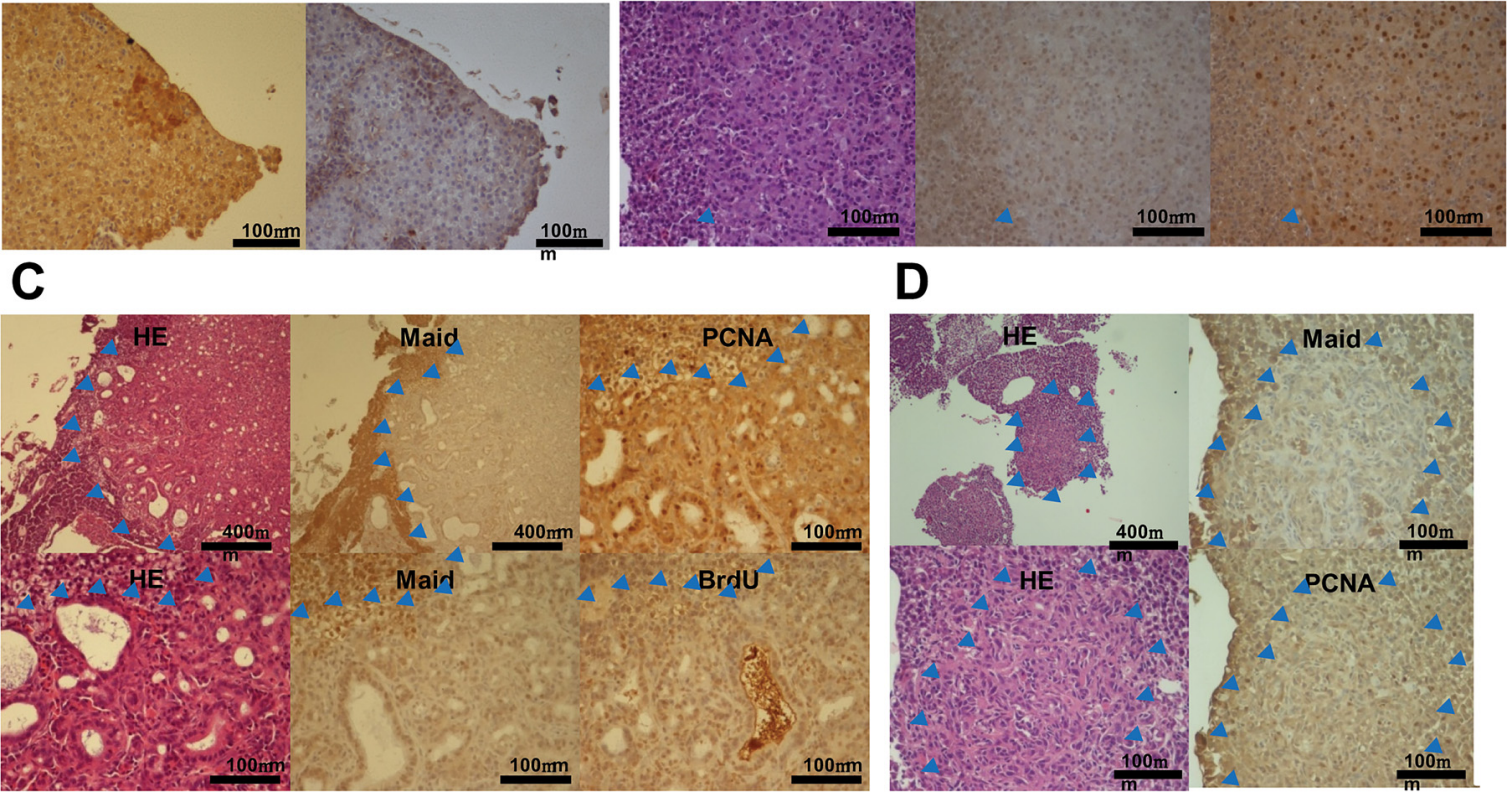
Immunohistochemical analysis of Maid expression during chemical carcinogenesis in zebrafish. (A) Liver samples from zebrafish that had been treated for 8 weeks with DEN and subsequently developed preneoplastic foci were subjected to immunohistochemical staining. Results are representative of 11 preneoplastic foci. Red arrow heads indicates Maid overexpressed lesion. Blue arrow heads indicate boundaries of tumor lesions. (B) Liver samples from zebrafish exhibiting HCC after 8 weeks of DEN treatment were subjected toimmunohistochemical staining. Results are representative of 15 HCCs. Blue arrow heads indicate boundaries of tumor lesions. (C) Liver samples from zebrafish exhibiting cholangiocarcinoma after 8 weeks of DEN treatment were subjected to immunohistochemical staining. Results are representative of 4 cholangiocarcinomas. Blue arrow heads indicate boundaries of tumor lesions. (D) Liver samples from zebrafish exhibiting mixed tumors after 8 weeks of DEN treatment were subjected to immunohistochemical staining. Results are representative of 21 mixed tumors. Blue arrow heads indicate boundaries of tumor lesions.

We then examined Maid expression in specific tumor types. Most preneoplastic foci positive for both PCNA and BrdU exhibited elevated expression of Maid (Fig 5A). Moreover, Maid had appeared to translocate to the hepatocyte nucleus (Fig 1D right). In contrast in HCC, which is a tumor type associated with compression of surrounding tissue and a total loss of normal tubular architecture, we observed considerable nuclear localization of PCNA but Maid expression was reduced compared to normal liver (Fig 5B). Maid protein was also lower in cholangiocarcinomas with developed ductal structures (Fig 5C), and in mixed tumors in which biliary epithelial cells formed small ductules with irregular cords of small hepatocytes (Fig 5D). These data are consistent with the proposed tumor-suppressive role of Maid in the liver.

Group 1 contained 2 fish (4.3%) with adenoma/HCC. Group 2 contained 15 with adenoma/HCC (30%), 4 with cholangiocarcinoma (6%), and 13 with mixed tumors (26%) (Table 1). Neither of the 2 adenoma/HCCs isolated from Group 1 exhibited any change in Maid expression. Preneoplastic foci and vacuolated focus observed in Group1 and Group2 showed no change in Maid expression (Table 2). Maid expression pattern in mixed tumor compared to that in preneoplastic foci was significantly different (Table 2). Maid expression pattern in adenoma/HCC compared to that in preneoplastic foci showed a tendency toward decreased expression but did not reach statistical significance (p-value = 0.06). These results bolster our conclusion that Maid functions as a moderate tumor suppressor in zebrafish liver.

**Table 1:** Incidents in Livers of DEN-treated Zebrafish.

	Group1 DEN 3weeks			Group2 DEN 8weeks	
	positive	negative		positive	negative
Liver abnormality	17	29	**	45	5
Number of fish with liver foci	11	35		8	42
Number of fish with liver tumor	2	44	**	25	25
Number of fish with liver adenoma or HCC	2	44	*	15	35
Number of fish with liver cholangiocarcinoma	0	46		4	47
Number of fish with liver mixed tumor	0	46	**	13	37
Number of fish with vacuolated focus	2	44	**	12	38

#1 Mann-Whitney U test and Student’s t-test were performed to assess differences between Group1 (DEN for 3 weeks + clean water for 5 weeks) and Group2 (DEN for 8 weeks + clean water for 12 weeks)

DEN 3 weeks: DEN for 3 weeks + clean water for 5 weeks

DEN 8 weeks: DEN for 8 weeks + clean water for 12 weeks

*indicates p<0.05

**indicates p<0.01

DEN for 3 weeks + clean water for 5 weeks vs DEN for 8 weeks + clean water for 12 weeks

**Table 2:** Maid Protein Expression in Livers of DEN-treated Zebrafish.

	Group1:DEN 3weeks			#1	Group2:DEN 8weeks			#2
Maid expression	increased	unchanged	decreased		increased	unchanged	decreased	
Preneoplastic focus	4	3	4		2	2	4	
Tumor adenoma/HCC	0	2	0	**	0	3	12	ns
Tumor cholangiocarcinoma	0	0	0		0	0	4	ns
Tumormixed tumor	0	0	0		0	0	13	**
Vacuolated focus	0	2	0		0	12	0	ns

#1 Mann-Whitney U test and Student’s t-test were performed to assess differences between Group1 (DEN for 3 weeks + clean water for 5 weeks) and Group2 (DEN for 8 weeks + clean water for 12 weeks)

#2 Mann-Whitney U test was performed to assess differences between preneoplastic focus and each lesion in Group2 (DEN for 8 weeks + clean water for 12 weeks)

**DEN 3 weeks: DEN for 3 weeks + clean water for 5 weeks

nsDEN 8 weeks: DEN for 8 weeks + clean water for 12 weeks

**indicates p<0.01, ns indicates not significant

DEN for 3 weeks + clean water for 5 weeks vs DEN for 8 weeks + clean water for 12 weeks

### Decreased liver cell proliferation in DEN-treated Maid transgenic medaka

**Fig 6:**
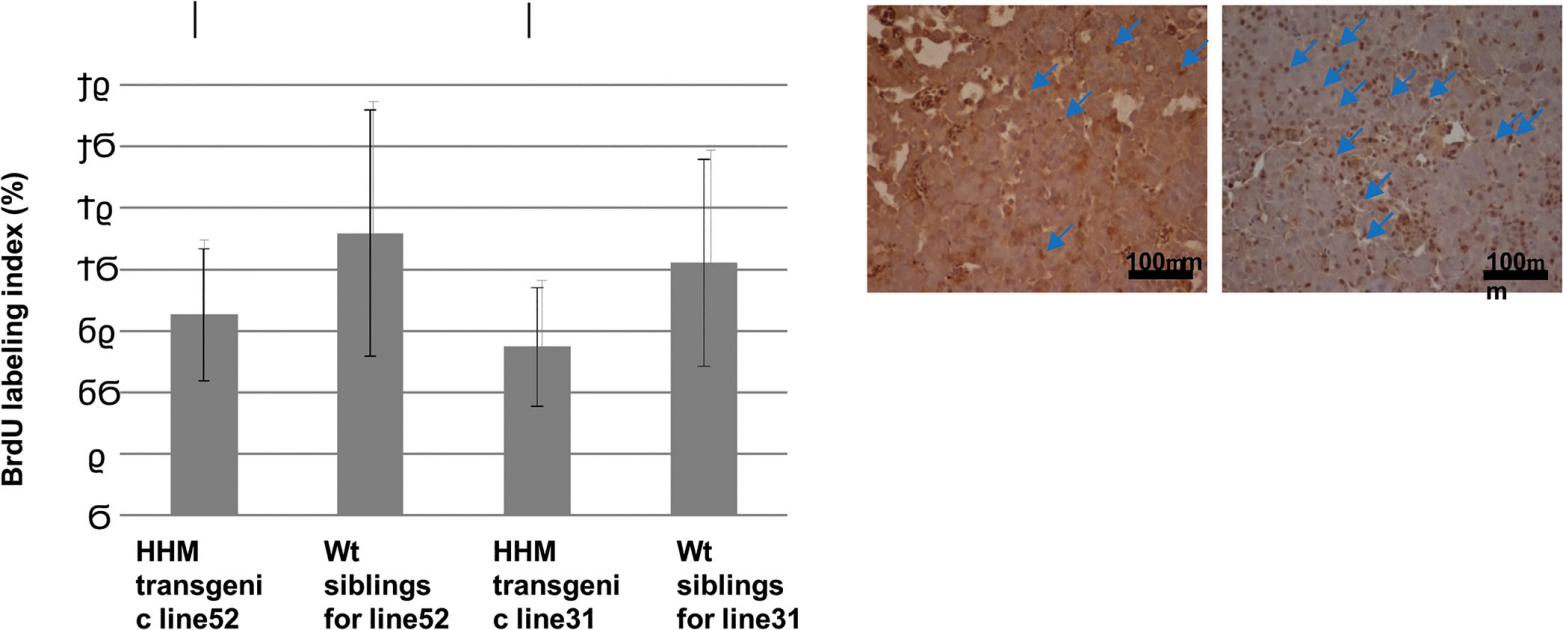
Characterization of Maid expression in transgenic medaka. (A) Western blot analysis of Maid (HHM) protein in various transgenic medaka lines overexpressing HHM-GFP. C, control non-transfected medaka. GFP, medaka transfected with control GFP plasmid. Results are representative of three independent experiments. (B) Immunohistochemical staining to detect BrdU in livers of the indicated lines of HHM transgenic medaka treated for 3 weeks with DEN and assayed at 6 months after DEN treatment. (C) Quantitation of the BrdU labeling results for the livers in (B). Arrows indicate BrdU positive cells. Results are the mean ± SD (n = 10 fish/group). *, p<0.05.

Medaka have long been used for cancer research, and many mutants are available. We generated transgenic medaka that ubiquitously overexpressed HHM using the sea bream β-actin promoter driving both HHM and EGFP expression. Among the several transgenic strains generated, line 52 exhibited the highest HHM expression, while lines 8, 22 and 31 exhibited lower HHM protein levels (Fig 6A). Lines 52 and 31 were chosen for further study. Sibling embryos that were homozygous for two copies of the HHM transgene [HHM Tg (+/+)], or heterozygous for one copy of the transgene [HHM Tg (+/-)], or non-transgenic siblings [HHM Tg (-/-)] were examined using the method of Brennan et al., who previously reported that transient chemically-induced hepatocyte proliferation in medaka can be assessed using BrdU incorporation [16]. We detected an increase in BrdU positive cells in line 52 and line 31 HHM Tg medaka at 7 days post-DEN administration. We then used BrdU incorporation and immunohistochemistry to detect changes in liver cell proliferation in HHM Tg medaka subjected to DEN exposure for 3 weeks (Fig 6B). The BrdU labeling index was significantly lower in line 52 and line 31 HHM Tg medaka than in their respective control non-transgenic HHM Tg siblings (Fig 6C). Thus, DEN-treated medaka overexpressing Maid proliferate less vigorously than DEN-treated WT medaka, suggesting that Maid also has an anti-tumor effect in this species.

### HHM transgenic medaka show delayed growth, decreased daily movement and a hunched back

**Fig 7:**
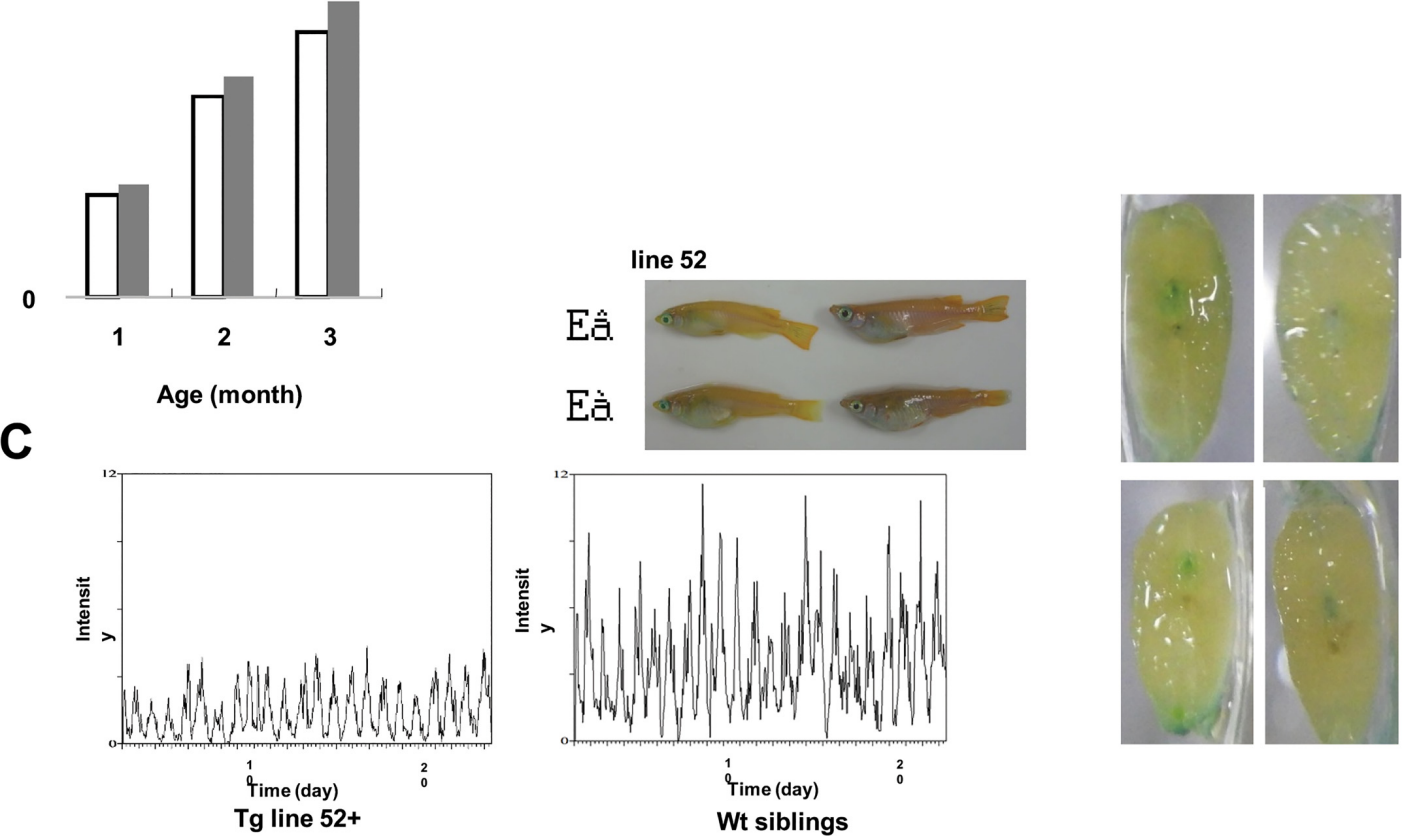
Aging is accelerated in HHM transgenic medaka. (A) The body lengths of line 52 HHM Tg medaka and control non-transgenic Tg siblings were measured after 1, 2 or 3 months rearing under standard conditions. *, p<0.05. (B) Differential appearances of the line 52 HHM Tg medaka (red arrows) and their non-transgenic siblings (black arrows) examined in (A) at one year after birth, shown both in (top) and out (bottom) of the water. Line 52 HHM Tg medaka at one year of age exhibited the prominently hunched back. (C) Representative actogram of a Line 52 HHM Tg medaka and their non-transgenic siblings. (Top) non-transgenic siblings (bottom) line 52 HHM Tg medaka (D) SA-β-galactosidase staining of cross-sections of the bodies of line 52 HHM Tg and non-transgenic Tg medaka siblings. Black arrows indicate the spinal cord. Results are representative of 5 fish examined per group.

We noticed that untreated HHM transgenic medaka allowed to reach maturity tended to have a shorter body length than non-transgenic siblings. To confirm this phenotype, siblings born on the same day were reared in a single aquarium and body length was formally measured at one, two and three months after fertilization. Line 52 HHM Tg medaka were significantly smaller in body length than their non-transgenicsiblings at 2 months and 3 months after birth (Fig 7A). HHM expression was still seen in 1 year old transgenic lines (S3 Fig). No other obvious differences from WT medaka were observed in either transgenic line up to the age of 10 months. However, when HHM Tg medaka were examined at approx. 12 months of age, many line 52 HHM Tg medaka exhibited a bent “hunched back” phenotype that was rarely seen in non-transgenic siblings of the same age (Fig 7B). This phenotype first appeared around 10 months after birth in both line 52 and line 31 HHM Tg medaka, and was observed in about 80% of line 52 HHM Tg fish at one year after birth. Furthermore, we evaluated movement of Tg medaka with chronobiology kit, and found that line 52 HHM Tg medaka showed decreased movement (Fig 7C). In hematoxylin and eosin-stained sections of the trunks of these fish, there were no obvious morphological changes (such as atrophy) to the musculature. However, we did observe eosinophilic deposits in the spinal cord cells of both lines of HHM Tg medaka that were absent from the spinal cord cells of non-transgenic siblings (S4 Fig), implicating the degeneration of these cells as a possible cause of the spinal abnormality. Furthermore, senescence-associated β-galactosidase staining in the spinal cord cells of HHM transgenic medaka was stronger than in non-transgenic siblings (Fig 7D), suggesting that overexpression of Maid accelerates aging in the spinal cord.

### Expression of Maid in a zebrafish liver cell line

To study effects of Maid overexpression in cells, analysis was run using liver-derived cultured cell lines, ZFL. We made ZFL harboring Maid-GFP overexpression vector. Maid-GFP fusion protein was present mainly in the cytoplasm, whereas GFP alone was distributed in both the nucleus and cytoplasm. Studying the proliferative capacity of Maid-GFP stable transformants revealed a slight growth inhibition compared to cells expressing GFP alone (S5 Fig). Furthermore, we performed microarray analysis to investigate gene expression in Maid overexpressing cells. Some interesting changes in up-regulated factors were determined in microarray analysis of ZFL overexpressing Maid. (S1 Table). We focused on TGF-beta as up-regulated factors and showed knowledge-based interaction network of TGF-beta targets after Maid overexpression in ZFL (S6 Fig).

## Discussion

In this study, we took advantage of two small fish models to extend the previously known physiological functions of Maid. Our amino acid sequence analysis revealed that Maid’s leucine zipper domain, which participates in protein-protein interactions, is highly conserved in zebrafish (S1 and S2 Figs). In addition, although some previous studies have described the abundance of *Maid* mRNA in specific tissues, our report is the first to investigate tissue-specific protein levels of this transcription factor. Upon examination of Maid protein in various zebrafish organs, we found that the highest levels of this molecule occur in the liver. Previous reports have shown that, upon translocation to the mammalian cell nucleus, Maid appears to exert cytostatic effects via interaction with molecules that are involved in cellular proliferation [18–20]. We observed that Maid was localized mainly in the cytoplasm of zebrafish hepatocytes, perhaps reflecting the regenerative potential of this organ. We speculate that Maid’s inhibitory effects on proliferation may be initially restrained by its cytoplasmic localization in order to permit hepatocyte proliferation during regeneration following liver damage, but that Maid can rapidly shuttle to the nucleus when needed to later prevent excessive proliferation. However, we acknowledge that Maid interacts with many other molecules in the nucleus and cytoplasm, and may therefore control cellular functions other than proliferation.

To date, there have been no reports on patterns of Maid protein expression during embryogenesis. Since small fish are highly suitable for vertebrate developmental analysis, we used zebrafish for this purpose. We found that unfertilized zebrafish eggs contain maternal-derived Maid protein, and that Maid levels increase following fertilization and development. The transient but robust reduction in Maid protein by knock-down had no effects, suggesting that continuous expression of Maid is dispensable for normal development. Although we saw no obvious defects in zebrafish development following Maid knock-down, we did observe increased expression of cyclin D1 at 16 dpf and 24 dpf. This result is consistent with a previous report in which overexpression of GCIP/Maid suppressed cyclin D1 promoter activity, whereas GCIP/Maid knockdown upregulated cyclin D1 expression [3]. To fully understand the effects of loss of Maid in zebrafish, a genetic knock-out study rather than morpholino knock-down will be required.

The mechanisms responsible for inhibiting hepatocyte proliferation for completing liver regeneration remain elusive. One candidate mechanism to regulate this process is the cytokine TGF-β because *TGF-β1* mRNA increases in the first 3–4 h after PH and plateaus after 3 days [21]. We have previously reported that *Maid* mRNA expression increases transiently after PH in rats [2]. Consistent with this finding, *Maid* mRNA had increased by day 1 after PH in zebrafish, with a return to normal levels after 7 days. Although Maid mRNA had increased at day1 after PH, there were no statistical significances in Maid protein expression level. We speculate that sensitivity of RT-PCR for change of Maid expression is better than that of protein. We also speculate that turnover rate of Maid was increased, because Maid is known to be degraded by proteasome system quickly [20]. Maid is a synexpression group-restricted regulator of TGF-β [22]. It is therefore possible that the increase in Maid induced by PH suppresses TGF-β signaling and causes it to plateau, decelerating regeneration. Thus, while the cytostatic activity of Maid may play a significant role in the suppression of liver regeneration, the mechanisms involved are likely distinct from those mediated by p21.

With respect to fin regeneration following amputation, no increase in *Maid* mRNA expression was observed during this process, perhaps reflecting the distinctly different modes of regeneration occurring in these two tissues. While simple duplication of mature hepatocytes in the remaining liver compensates for the lost tissue in post-PH liver, fin regeneration requires mature fin cells to de-differentiate and form blastemas[23].

In addition to its proposed role during liver regeneration, Maid has been considered a putative tumor suppressor gene. The onset of tumorigenesis is suppressed in transgenic mice overexpressing Maid [6], and liver cancer occurs more frequently in Maid knockout mice than in WT controls [7]. In our short-term *in vivo* study of zebrafish, 4/11 (36%) of the preneoplastic foci induced by DEN administration showed elevated Maid protein. It is possible that this increase in Maid expression is an antiproliferative response to DEN treatment that prevents full-blown tumorigenesis. However, the fact that Maid expression was unchanged in 27% of preneoplastic foci and decreased in 36% of them suggests that Maid levels may be directly related to the degree of malignancy and differentiation of a transformed cell. Similar correlations have been found in the case of other tumor suppressors.

Maid is an HLH transcriptional regulator, a class of factor for which subcellular localization is important. In line with this requirement, we found Maid to be abundant in the cytoplasm of larval hepatocytes immediately after hatching, a time when active proliferation takes place. Maid was also abundant in the cytoplasm of hepatocytes of adult WT zebrafish. After DEN administration, however, Maid appeared to translocate to the hepatocyte nucleus, presumably to participate in growth-inhibitory signaling. Consistent with the importance of the nuclear localization of Maid for its tumor suppressor activity, it has recently been reported in human cancer cells that RRAD (ras associated with diabetes) antagonizes Maid’s anti-tumor role by facilitating Maid export from the nucleus to the cytoplasm. Furthermore, RRAD knockdown in human cancer cells results in cell cycle arrest and premature senescence associated with nuclear translocation of Maid [9].

Our study also examined the cytostatic properties of Maid in detail. To this end, we generated transgenic medaka overexpressing HHM and found that high levels of this protein suppressed the hepatocyte proliferation that normally follows DEN treatment. Since HHM is normally localized in the cytoplasm, it does not exhibit a strong cytostatic effect. However, we speculate that, upon potentially tumorigenic stresses such as DEN treatment, the nuclear translocation of HHM is stimulated and its cytostatic effects are manifested.

Although no changes to the liver or other obvious morphological changes were observed during development, HHM transgenic medaka had shorter body lengths than non-transgenic siblings and showed delayed growth. Systemic Maid overexpression is supposed to inhibit cell growth, but we did not detect any significant differences in the expression of cell cycle-related genes such as cyclin D1. This suggests that most of the HHM protein could be localized in the hepatocyte cytoplasm and could not exert its nuclear function. Although no 10-month-old HHM transgenic medaka exhibited an obvious senescence-like phenotype in any tissue analyzed, some of these fish developed a hunched back phenotype sometimes seen in very old WT medaka. While no changes to the musculature in the spinal region of HHM transgenic medaka were detected, deposition of intracellular granules was observed in the spinal cord, suggesting that neurodegeneration may have contributed to the bending of the trunk.

Maid has been previously linked to senescence in human cancer cell line [9]. We found that staining with SA-β-galactosidase, which is used as a senescence marker, was more intense in the spinal cords of HHM transgenic medaka, implying that senescence had accelerated. This is consistent with the report that RRAD knockdown in human cancer cells results in nuclear translocation of Maid and premature senescence [9]. We examined gene expression in ZFL cell lines by microarray analysis, and found that some important upstream regulators (S1 Table). Estrogen plays a very important role in a variety of biological aspects. It is reported that 17beta-E2 might relieve H2O2-induced mitochondrial damage through estrogen receptor and delay the vascular endothelial cell senescence. It is interesting to find TGF-beta as a upstream regulator, because it is related to senescence, and Maid is a synexpression group-restricted regulator of TGF-beta signalling. Furthermore, Maid can participate in a protein complex with SirT6[24], which is a histone H3 deacetylase involved in maintaining telomeres and chromosomes, repairing DNA, and controlling metabolism. SirT6 knockout mice die one month after birth due to premature aging-like symptoms, such as lordokyphosis. Thus, Maid may exert some of its senescence-promoting effects via functional inhibition of SirT6, a possibility currently under examination.

In conclusion, our work has demonstrated that Maid has cytostatic activity in non-mammalian vertebrates, and that nuclear translocation is likely required for this effect. A greater mechanistic understanding of this process may point toward potential strategies for activating Maid in order to inhibit tumor growth.

## Supporting Information

S1 Fig Sequence characterization and phylogenetic analysis of zebrafish Maid. Because initial BLAST data failed to identify Maid homologs in zebrafish, consensus sequences were derived from human and mouse Maid sequences to prepare primers. mRNA was isolated from zebrafish ovaries, which were expected to have the highest expression of *Maid* mRNA, and cDNA was synthesized to obtain a full-length zebrafish homologue of Maid (ZHM; GenBank, EF581817). The full-length cDNA contained 1210 bp encoding a 344 amino acid protein. (A) Comparison of amino acid sequences of Maid isolated from zebrafish and the indicated species. (*), identical amino acids; (:), highly conserved amino acids; (.), conserved amino acids as determined by Clustal Consensus analysis. HLH, helix-loop-helix motif; LZ, leucine zipper motif.
(PDF)

S2 Fig Phylogenetic tree of Maid. Phylogenetic tree based on ClustalW alignment generated with the TreeView program. The scale below the tree indicates an amino acid replacement distance of 0.1. Among vertebrates, zebrafish Maid was closest in sequence to that of the puffer fish and stickleback fish. At the amino acid level, zebrafish Maid is 38% homologous to human Maid, 48% homologous to chicken Maid, 43% to *Xenopus* Maid, 48% to tetraodon Maid, and 59% to stickleback Maid.
(PDF)

S3 Fig Western blotting analysis of Maid in 1 year old transgenic medaka. Protein samples were prepared from liver of transgenic medaka line 52 and Wt siblings at 1 year old. Lane 1–5: Wt sibling for line 52, Lane 6–11: HHM transgenic line 52.
(PDF)

S4 Fig HE staining of cross-sections of spinal cords. HE staining of cross-sections of spinal cords prepared from the indicated lines of HHM transgenic medaka. Red arrows indicate granular deposits observed only in individuals expressing HHM.
(PDF)

S5 Fig Analysis of zebrafish liver cell line (ZFL) overexpressing Maid. A: Immunofluorescence analysis of (left) ZFL cells expressing Maid-GFP and (right) control ZFL cells expressing GFP. B: Growth curve of the cells.
(PDF)

S6 Fig Gene expression profiling identified an association between Maid alterations and TGF-beta targets. Knowledge-based interaction network of TGF-beta targets after Maid overexpression in ZFL. The network was built based on the TGF-beta interactome in the Ingenuity IPA database overlaid with microarray data from ZFL overexpressing Maid 1.5-fold change cut-off. The intensity of the color indicates the degree of up- (red) or down- (green) regulation. TGF-beta is surrounded by blue line.
(PDF)

S1 Table Upstream regulators determined by microarray analysis. (XLS)

## References

[1] Hwang SY, Oh B, Fuchtbauer A, Fuchtbauer EM, Johnson KR, Solter D, et al Maid: a maternally transcribed novel gene encoding a potential negative regulator of bHLH proteins in the mouse egg and zygote. Dev Dyn. 1997;209(2):217–26. . 9186056 10.1002/(SICI)1097-0177(199706)209:2<217::AID-AJA7>3.0.CO;2-L

[2] Terai S, Aoki H, Ashida K, Thorgeirsson SS. Human homologue of maid: A dominant inhibitory helix-loop-helix protein associated with liver-specific gene expression. Hepatology. 2000;32(2):357–66. . 10915743 10.1053/jhep.2000.9092

[3] Xia C, Bao Z, Tabassam F, Ma W, Qiu M, Hua S, et al GCIP, a novel human grap2 and cyclin D interacting protein, regulates E2F-mediated transcriptional activity. J Biol Chem. 2000;275(27):20942–8. . 10801854 10.1074/jbc.M002598200

[4] Yao Y, Doki Y, Jiang W, Imoto M, Venkatraj VS, Warburton D, et al Cloning and characterization of DIP1, a novel protein that is related to the Id family of proteins. Exp Cell Res. 2000;257(1):22–32. . 10854051 10.1006/excr.2000.4884

[5] Takami T, Terai S, Yokoyama Y, Tanimoto H, Tajima K, Uchida K, et al Human homologue of maid is a useful marker protein in hepatocarcinogenesis. Gastroenterology. 2005;128(5):1369–80. . 15887118 10.1053/j.gastro.2005.03.014

[6] Ma W, Xia X, Stafford LJ, Yu C, Wang F, LeSage G, et al Expression of GCIP in transgenic mice decreases susceptibility to chemical hepatocarcinogenesis. Oncogene. 2006;25(30):4207–16. . 16501603 10.1038/sj.onc.1209450

[7] Sonnenberg-Riethmacher E, Wustefeld T, Miehe M, Trautwein C, Riethmacher D. Maid (GCIP) is involved in cell cycle control of hepatocytes. Hepatology. 2007;45(2):404–11. . 17256742 10.1002/hep.21461

[8] Chen WC, Su PF, Jin YT, Chang MC, Chang TW. Immunohistochemical expression of GCIP in breast carcinoma: relationship with tumour grade, disease-free survival, mucinous differentiation and response to chemotherapy. Histopathology. 2008;53(5):554–60. 10.1111/j.1365-2559.2008.03154.x 18983464

[9] Lee I, Yeom SY, Lee SJ, Kang WK, Park C. A novel senescence-evasion mechanism involving Grap2 and Cyclin D interacting protein inactivation by Ras associated with diabetes in cancer cells under doxorubicin treatment. Cancer research. 2010;70(11):4357–65. 10.1158/0008-5472.CAN-09-3791 . 20460530

[10] Amatruda T, Institute for Clinical Systems Improvement. Technology Assessment Committee. Genetic testing for hereditary nonpolyposis colorectal cancer (HNPCC) Bloomington, MN: ICSI; 2002. 13 p. p.

[11] Au DW, Mok HO, Elmore LW, Holt SE. Japanese medaka: a new vertebrate model for studying telomere and telomerase biology. Comp Biochem Physiol C Toxicol Pharmacol. 2009;149(2):161–7. 10.1016/j.cbpc.2008.08.005 . 18790082

[12] Goessling W, North TE, Zon LI. New waves of discovery: modeling cancer in zebrafish. J Clin Oncol. 2007;25(17):2473–9. 10.1200/JCO.2006.08.9821 . 17557959

[13] Fujisawa K, Murakami R, Horiguchi T, Noma T. Adenylate kinase isozyme 2 is essential for growth and development of Drosophila melanogaster. Comp Biochem Physiol B Biochem Mol Biol. 2009;153(1):29–38. 10.1016/j.cbpb.2009.01.006 . 19416704

[14] Fujisawa K, Terai S, Hirose Y, Takami T, Yamamoto N, Sakaida I. Senescence marker protein 30 (SMP30)/regucalcin (RGN) expression decreases with aging, acute liver injuries and tumors in zebrafish. Biochemical and biophysical research communications. 2011;414(2):331–6. 10.1016/j.bbrc.2011.09.067 . 21951853

[15] Sadler KC, Krahn KN, Gaur NA, Ukomadu C. Liver growth in the embryo and during liver regeneration in zebrafish requires the cell cycle regulator, uhrf1. Proc Natl Acad Sci U S A. 2007;104(5):1570–5. . 17242348 10.1073/pnas.0610774104 PMC1785278

[16] Brennan LM, Boncavage-Hennessey EM, Wolfe MJ, Toussaint MW, Dennis WE, Rosencrance AB, et al An in vivo method for using 5-bromo-2'-deoxyuridine (BrdU) as a marker of chemically-induced hepatocellular proliferation in the Japanese medaka (Oryzias latipes). Toxicol Pathol. 2001;29(3):387–97. . 11442025 10.1080/019262301316905354

[17] Nasevicius A, Ekker SC. Effective targeted gene 'knockdown' in zebrafish. Nat Genet. 2000;26(2):216–20. . 11017081 10.1038/79951

[18] Chang MS, Chang CL, Huang CJ, Yang YC. p29, a novel GCIP-interacting protein, localizes in the nucleus. Biochemical and biophysical research communications. 2000;279(2):732–7. . 11118353 10.1006/bbrc.2000.3992

[19] Chang TW, Chen CC, Chen KY, Su JH, Chang JH, Chang MC. Ribosomal phosphoprotein P0 interacts with GCIP and overexpression of P0 is associated with cellular proliferation in breast and liver carcinoma cells. Oncogene. 2008;27(3):332–8. . 17621266 10.1038/sj.onc.1210651

[20] Chellas-Gery B, Linton CN, Fields KA. Human GCIP interacts with CT847, a novel Chlamydia trachomatis type III secretion substrate, and is degraded in a tissue-culture infection model. Cellular microbiology. 2007;9(10):2417–30. . 17532760 10.1111/j.1462-5822.2007.00970.x

[21] Braun L, Mead JE, Panzica M, Mikumo R, Bell GI, Fausto N. Transforming growth factor beta mRNA increases during liver regeneration: a possible paracrine mechanism of growth regulation. Proc Natl Acad Sci U S A. 1988;85(5):1539–43. 3422749 10.1073/pnas.85.5.1539 PMC279808

[22] Ikushima H, Komuro A, Isogaya K, Shinozaki M, Hellman U, Miyazawa K, et al An Id-like molecule, HHM, is a synexpression group-restricted regulator of TGF-beta signalling. Embo J. 2008;27(22):2955–65. 10.1038/emboj.2008.218 18923419 PMC2570476

[23] Albrecht JH, Poon RY, Ahonen CL, Rieland BM, Deng C, Crary GS. Involvement of p21 and p27 in the regulation of CDK activity and cell cycle progression in the regenerating liver. Oncogene. 1998;16(16):2141–50. 10.1038/sj.onc.1201728 . 9572495

[24] Michishita E, McCord RA, Berber E, Kioi M, Padilla-Nash H, Damian M, et al SIRT6 is a histone H3 lysine 9 deacetylase that modulates telomeric chromatin. Nature. 2008;452(7186):492–6. 10.1038/nature06736 18337721 PMC2646112

