# Correction: Evidence for a Role of the Transcriptional Regulator Maid in Tumorigenesis and Aging

**DOI:** 10.1371/journal.pone.0137156

**Published:** 2015-08-25

**Authors:** Koichi Fujisawa, Shuji Terai, Toshihiko Matsumoto, Taro Takami, Naoki Yamamoto, Hiroshi Nishina, Makoto Furutani-Seiki, Isao Sakaida

There are errors in Figs 1–7. Potions of the images are omitted in error. Please view the correct Figs 1–7 here.

**Fig 1:**
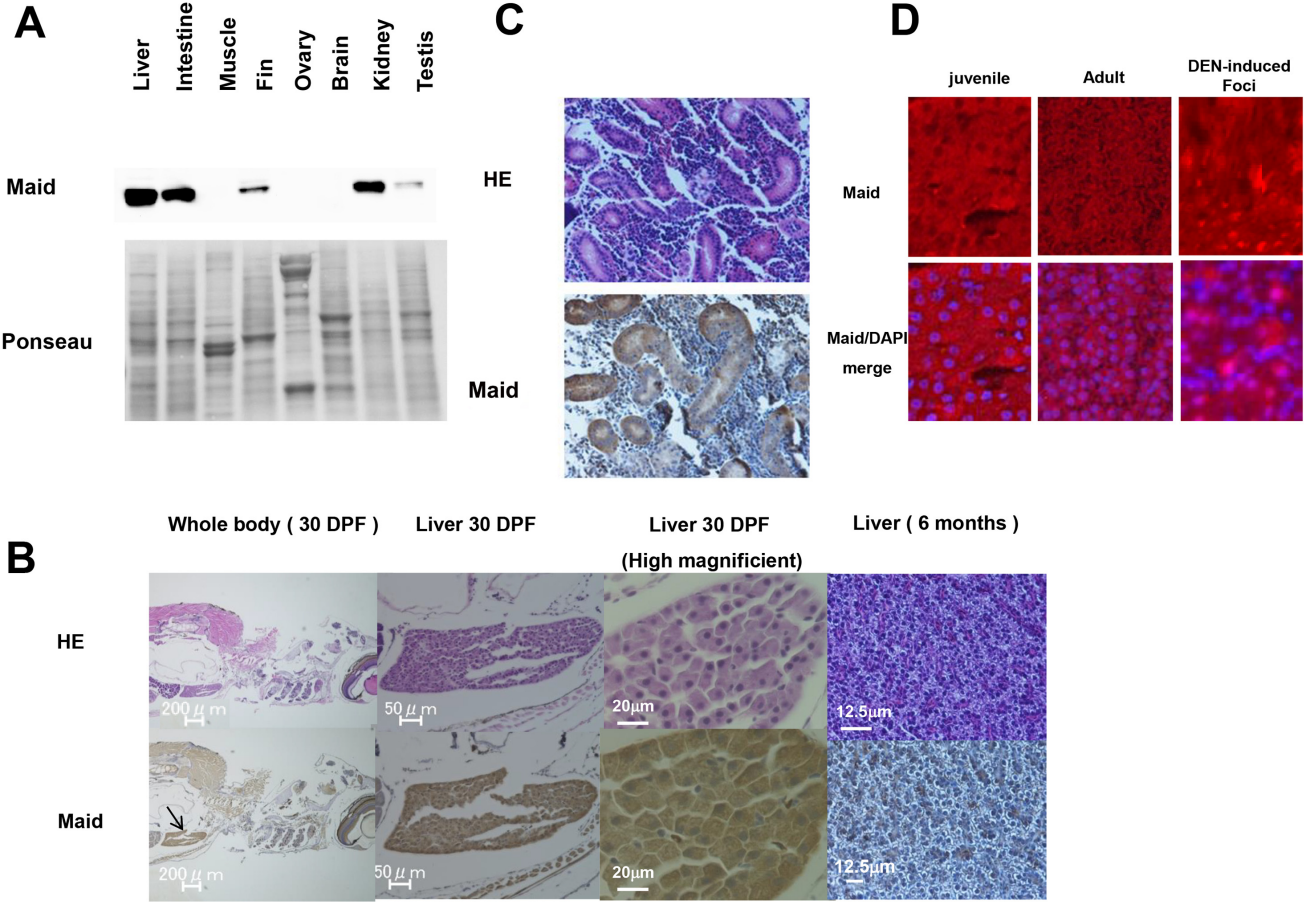
Zebrafish Maid expression in adult tissues. (A) Western blot to detect Maid in total protein isolated from the indicated tissues of adult zebrafish. Ponseau-S staining shows total protein bands. (B) Top: Hematoxylin and eosin (HE) staining of the whole body and liver of a WT zebrafish embryo at 30 dpf, and the liver of a WT adult zebrafish at 6 months of age. Bottom: Immunohistochemical staining to detect Maid in the zebrafish tissues in the top panel. Black arrow indicates the liver, where the highest level of Maid expression was observed. (C) Top: HE staining of zebrafish kidney at 6 months of age. Bottom: Immunohistochemical staining to detect Maid in the zebrafish kidney in the top panel. (D) Localization of Maid in zebrafish liver. Immunofluorescent analysis of Maid protein localization in untreated juvenile zebrafish liver, adult zebrafish liver, and a DEN-induced foci. Red, anti-zebrafish Maid antibody; blue, DAPI (visualization of nuclei).

**Fig 2:**
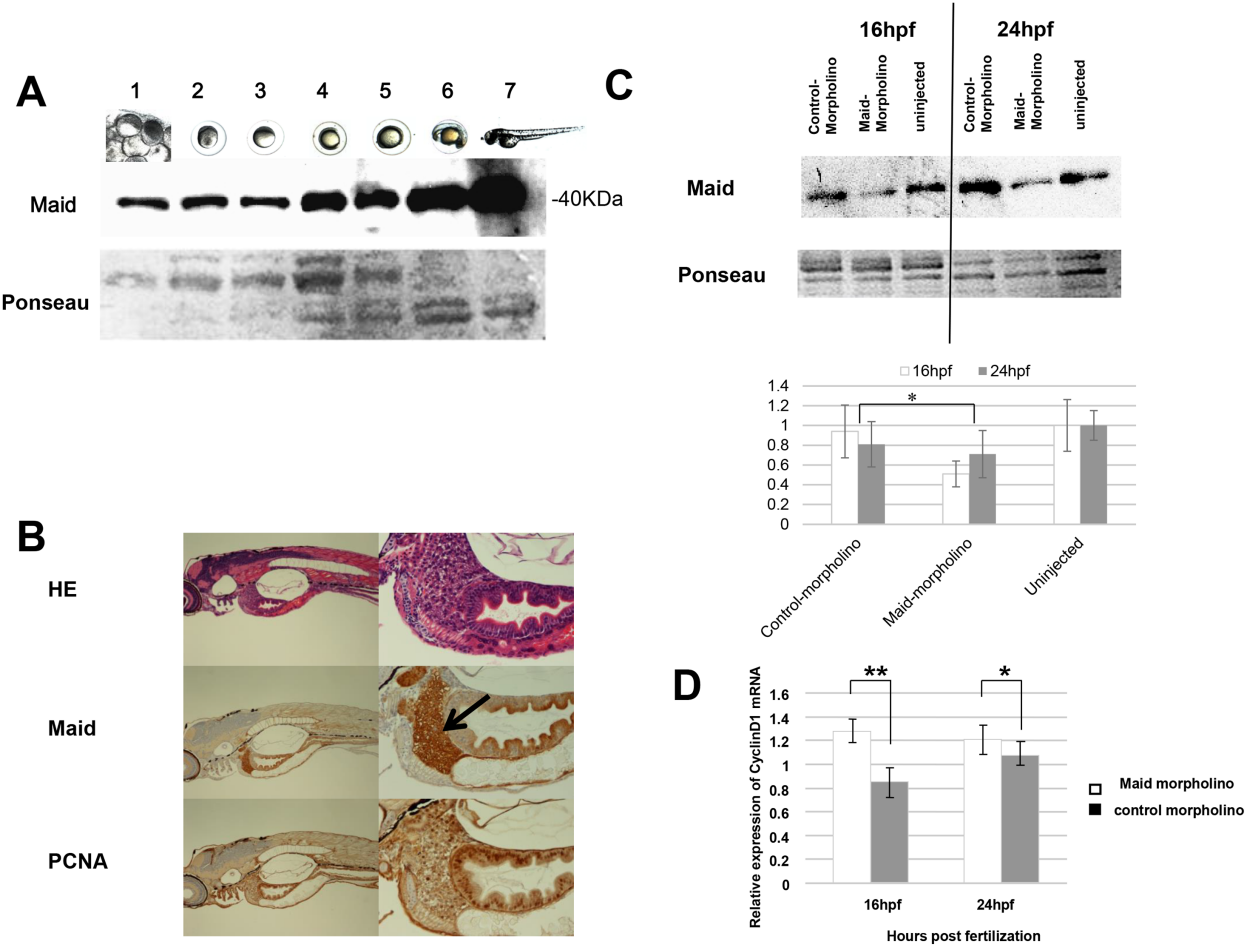
Maid expression during zebrafish development. (A) Western blot analysis of Maid expression: lane 1, ovary; 2, unfertilized egg (0 h); 3, cleavage (1.5 h); 4, blastula (4 h); 5, gastrula (10 h); 6, segmentation (20 h), and 7, hatching (72 h). Ponseau staining as a loading control. Macroscopic views of developing zebrafish embryos are also shown. (B) HE staining of whole zebrafish at the hatching stage shown at low (left) and high (right) magnifications. Black arrow indicates the liver. (C) Top: Fertilized zebrafish eggs were left uninjected (control, lane 3), or microinjected at the one- to two-cell stage with control morpholino (lane 1) or morpholino against Maid (ZHM) (lane 2). Middle: Western blot analysis was performed to detect Maid in protein isolated from these embryos at 16 hpf or 24 hpf. Results are representative of 20 embryos examined per treatment. Bottom: Graphical representation of Western blot analysis, *, p<0.05. (D) Real-time RT-PCR analysis of *cyclin D1* mRNA expression in extracts of the treated zebrafish embryos in (C). Results are the mean ± SD (n = 20/group) and are expressed relative to levels of *El1a* mRNA (set to 1 as internal control). *, p<0.05; **, p<0.01.

**Fig 3:**
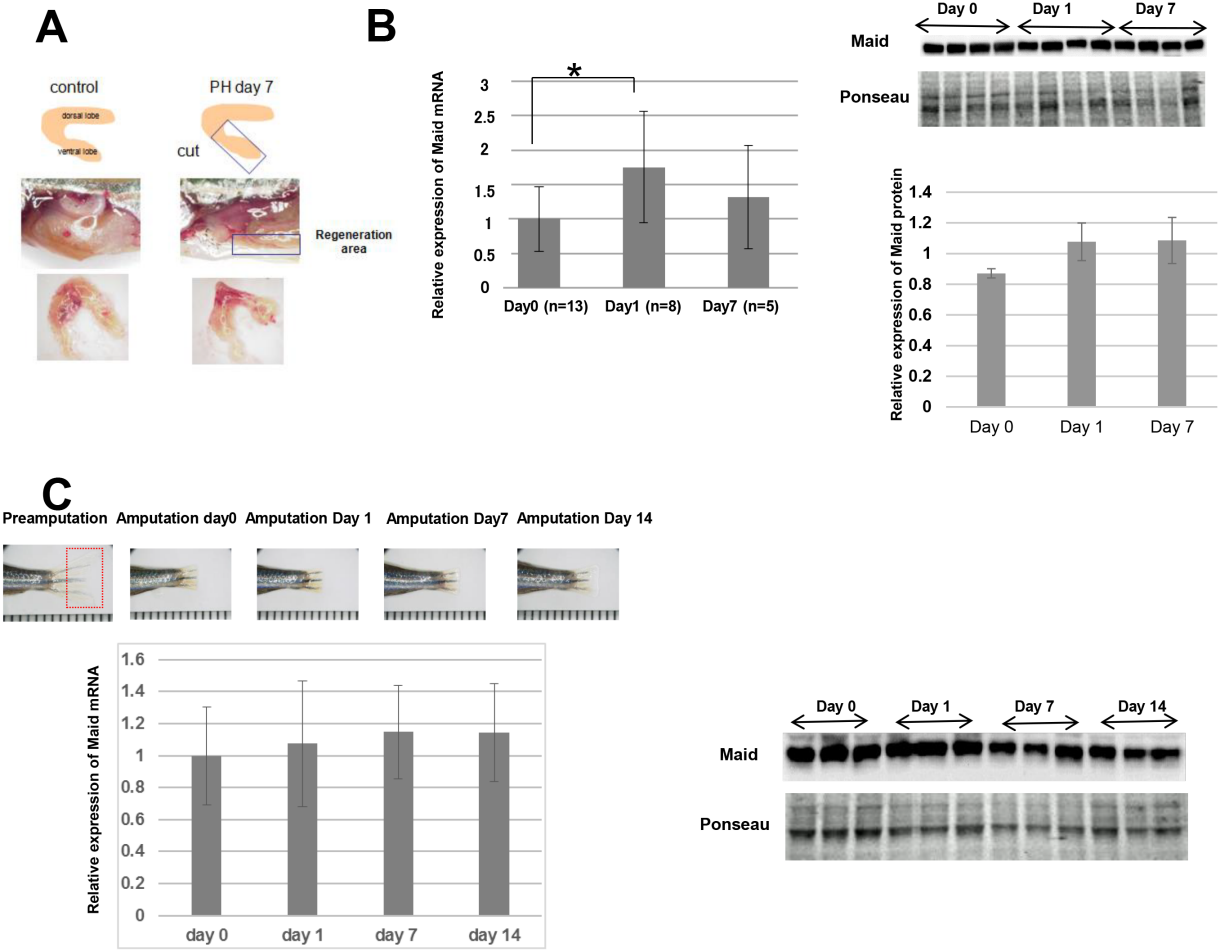
Maid expression following partial hepatectomy or fin amputation in zebrafish. (A) Top: Diagram of the area of adult zebrafish liver subjected to PH. Middle: Macroscopic views of fish abdomens, sliced in the middle. About 30% of the ventral lobe of the liver was removed. The area of the liver regeneration is demarcated by a rectangle. Bottom: Macroscopic view of the livers in the middle panel after 7 days of regeneration. Results are representative of 10 fish per group. (B) Left: RT-PCR analysis of *Maid* mRNA expression in regenerated zebrafish liver at the indicated times after PH. Results are the mean ± SD expressed relative to Gapdh and are representative of at least 5 fish per group, *, p<0.05. Right: Western blotting analysis of Maid in regenerated zebrafish liver. (C) Left Top: Macroscopic views of a representative adult zebrafish tail fin before and after amputation. Fin regeneration was monitored for 14 days. Left Bottom: RT-PCR analysis of *Maid* mRNA expression in regenerating fin at the indicated times after fin amputation. Results are the mean ± SD (n = 3/group) and are expressed relative to *El1a* mRNA in fish at Pre-Amputation. Right: Western blotting analysis of Maid in regenerating fin.

**Fig 4:**
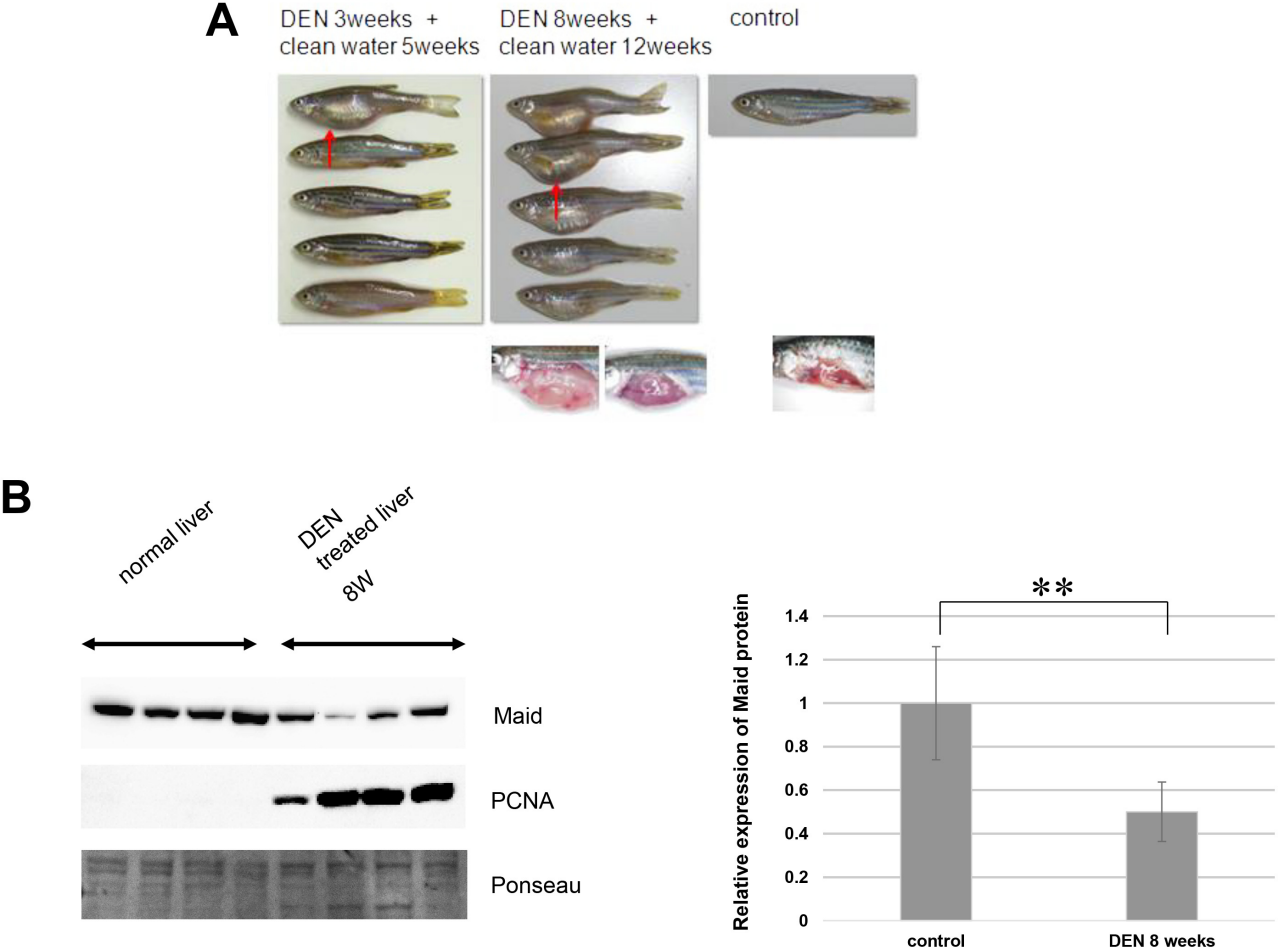
Liver expansion and reduced Maid expression during chemical carcinogenesis in zebrafish. (A) Top left: Macroscopic views of zebrafish in Group 1. Red arrow indicates a distended abdomen. Top middle: Macroscopic views of zebrafish in Group 2. Top right: Macroscopic view of a control zebrafish reared for 5 weeks in clean water and not exposed to DEN. Bottom panels show macroscopic views of (middle) livers of two DEN-treated zebrafish in Group 2 and (right) healthy control liver. (B) Western blot analysis of Maid and PCNA protein in two normal zebrafish livers and livers of 5 zebrafish subjected to 8 weeks of DEN administration. Left: Graphical representation of Western blot analysis, **, p<0.01.

**Fig 5:**
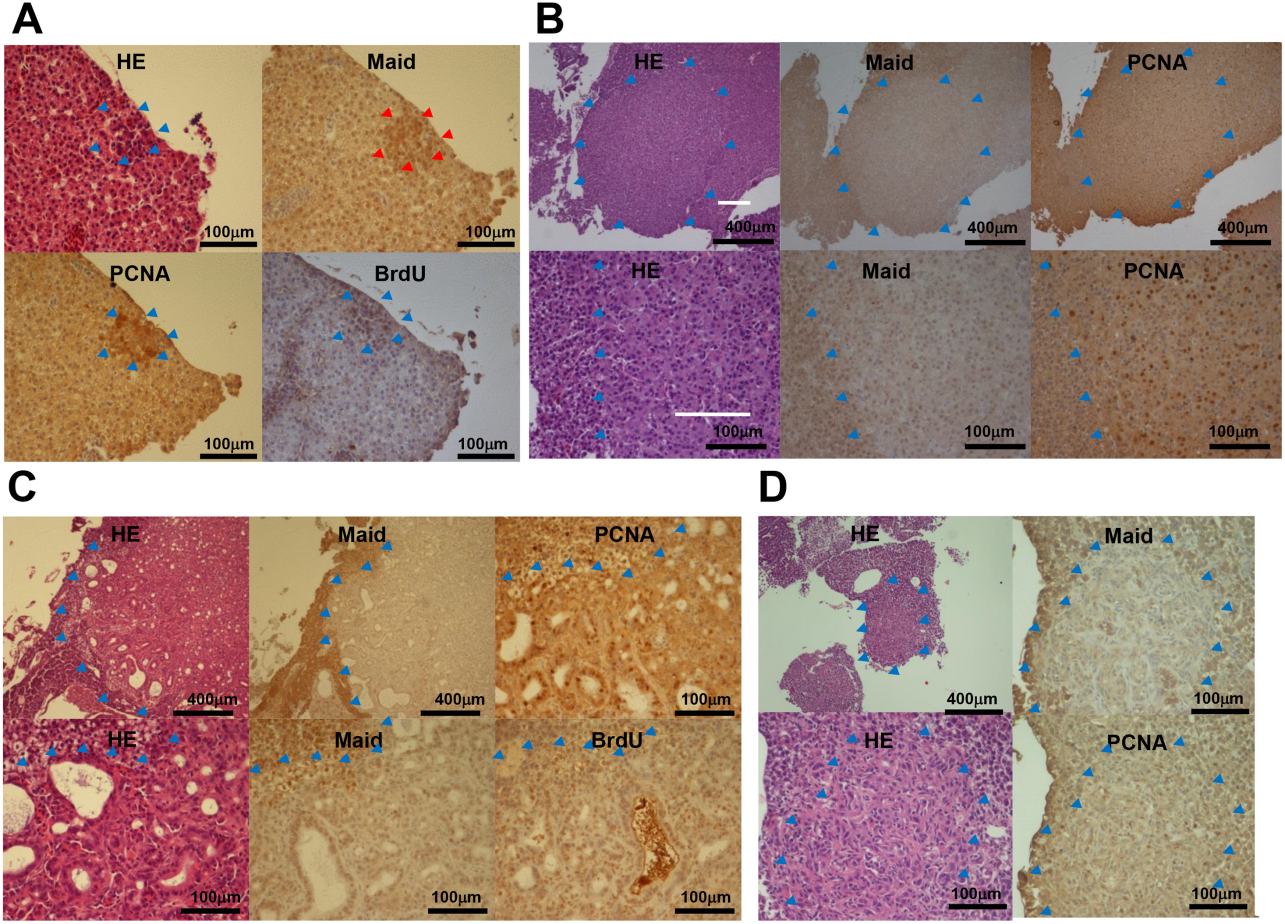
Immunohistochemical analysis of Maid expression during chemical carcinogenesis in zebrafish. (A) Liver samples from zebrafish that had been treated for 8 weeks with DEN and subsequently developed preneoplastic foci were subjected to immunohistochemical staining. Results are representative of 11 preneoplastic foci. Red arrow heads indicates Maid overexpressed lesion. Blue arrow heads indicate boundaries of tumor lesions. (B) Liver samples from zebrafish exhibiting HCC after 8 weeks of DEN treatment were subjected toimmunohistochemical staining. Results are representative of 15 HCCs. Blue arrow heads indicate boundaries of tumor lesions. (C) Liver samples from zebrafish exhibiting cholangiocarcinoma after 8 weeks of DEN treatment were subjected to immunohistochemical staining. Results are representative of 4 cholangiocarcinomas. Blue arrow heads indicate boundaries of tumor lesions. (D) Liver samples from zebrafish exhibiting mixed tumors after 8 weeks of DEN treatment were subjected to immunohistochemical staining. Results are representative of 21 mixed tumors. Blue arrow heads indicate boundaries of tumor lesions.

**Fig 6:**
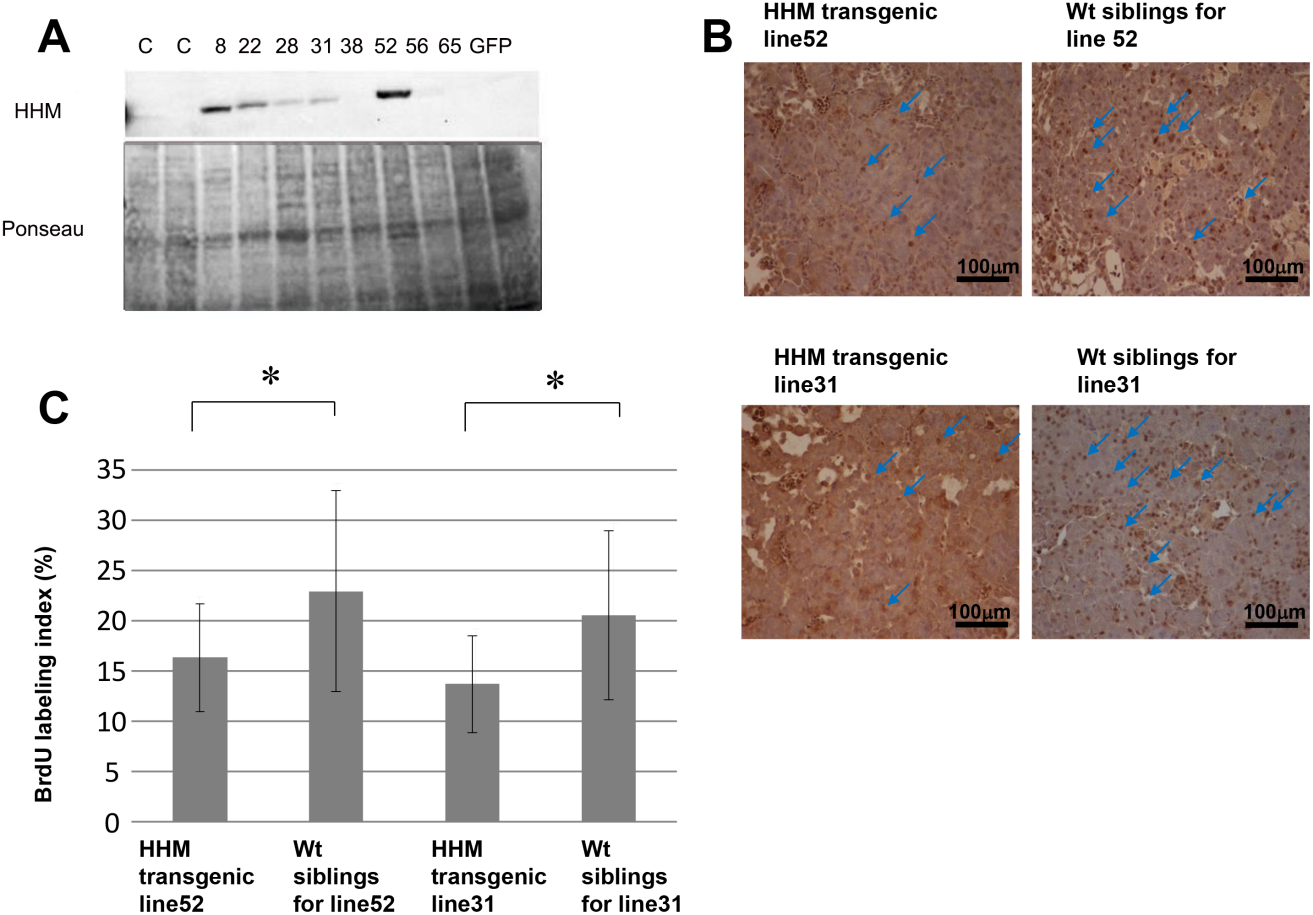
Characterization of Maid expression in transgenic medaka. (A) Western blot analysis of Maid (HHM) protein in various transgenic medaka lines overexpressing HHM-GFP. C, control non-transfected medaka. GFP, medaka transfected with control GFP plasmid. Results are representative of three independent experiments. (B) Immunohistochemical staining to detect BrdU in livers of the indicated lines of HHM transgenic medaka treated for 3 weeks with DEN and assayed at 6 months after DEN treatment. (C) Quantitation of the BrdU labeling results for the livers in (B). Arrows indicate BrdU positive cells. Results are the mean ± SD (n = 10 fish/group). *, p<0.05.

**Fig 7:**
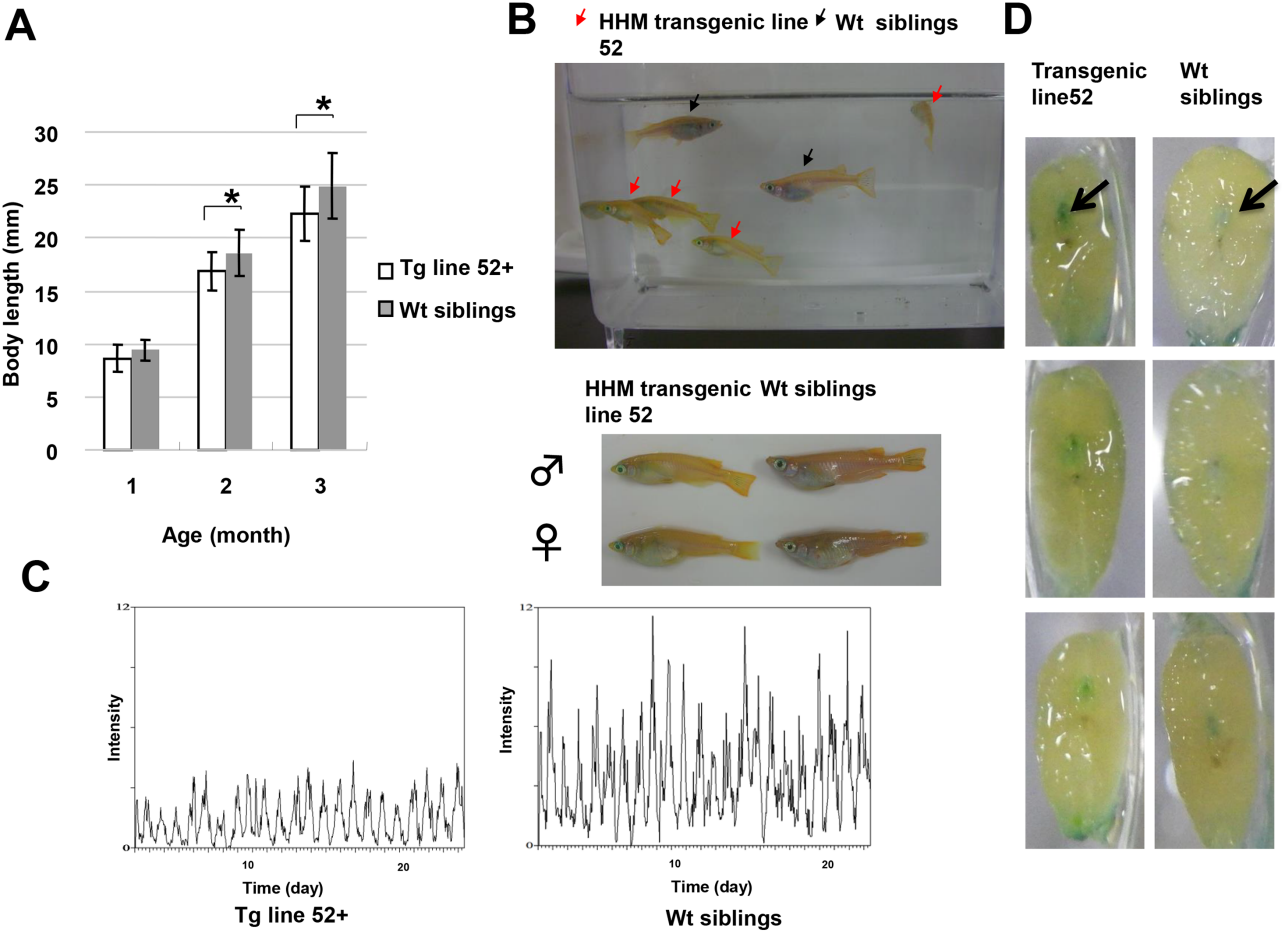
Aging is accelerated in HHM transgenic medaka. (A) The body lengths of line 52 HHM Tg medaka and control non-transgenic Tg siblings were measured after 1, 2 or 3 months rearing under standard conditions. *, p<0.05. (B) Differential appearances of the line 52 HHM Tg medaka (red arrows) and their non-transgenic siblings (black arrows) examined in (A) at one year after birth, shown both in (top) and out (bottom) of the water. Line 52 HHM Tg medaka at one year of age exhibited the prominently hunched back. (C) Representative actogram of a Line 52 HHM Tg medaka and their non-transgenic siblings. (Top) non-transgenic siblings (bottom) line 52 HHM Tg medaka (D) SA-β-galactosidase staining of cross-sections of the bodies of line 52 HHM Tg and non-transgenic Tg medaka siblings. Black arrows indicate the spinal cord. Results are representative of 5 fish examined per group.
