# Supplementary figures and images for: Evidence for a Role of the Transcriptional Regulator Maid in Tumorigenesis and Aging
Source: PLoS One. 2015 Jun 24;10(6):e0129950. doi: 10.1371/journal.pone.0129950 (PMC4479567; doi:10.1371/journal.pone.0129950)

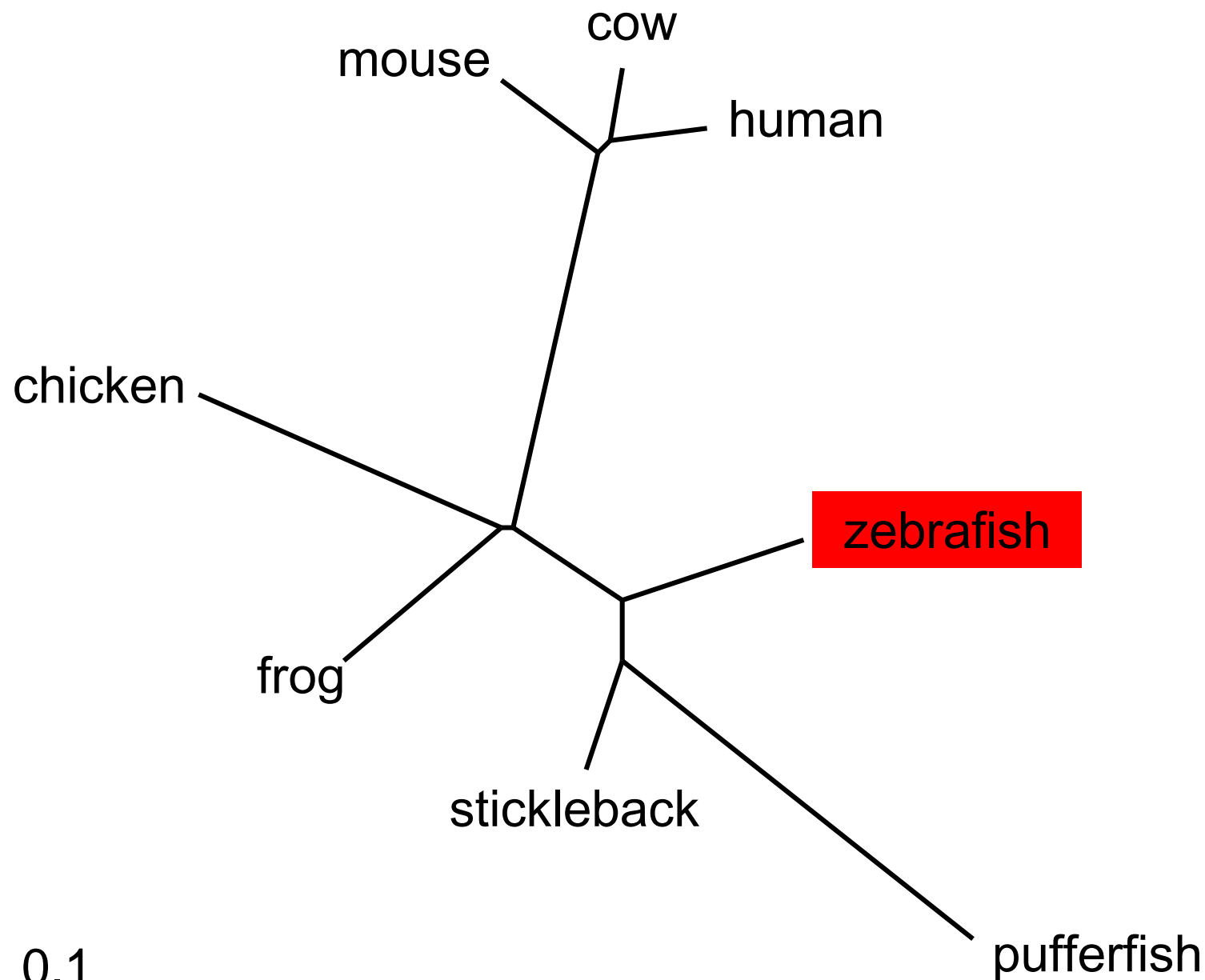

Supplement: S2 Fig — Phylogenetic tree based on ClustalW alignment generated with the TreeView program. The scale below the tree indicates an amino acid replacement distance of 0.1. Among vertebrates, zebrafish Maid was closest in sequence to that of the puffer fish and stickleback fish. At the amino acid level, zebrafish Maid is 38% homologous to human Maid, 48% homologous to chicken Maid, 43% to Xenopus Maid, 48% to tetraodon Maid, and 59% to stickleback Maid. (PDF) [file pone.0129950.s002.pdf]

Wt siblings for line52

HHM transgenic line52

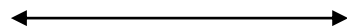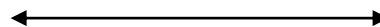

Maid

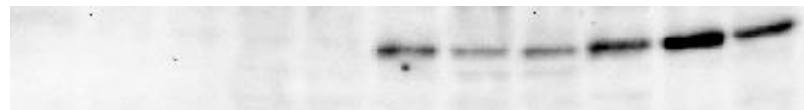

Ponseau

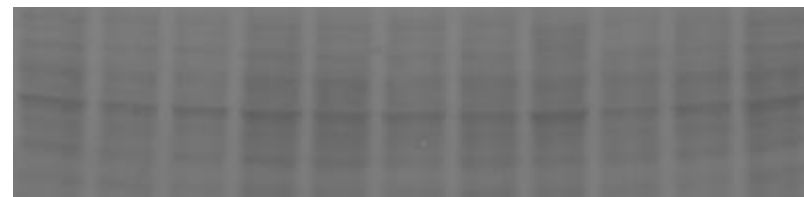

Supplement: S3 Fig — Protein samples were prepared from liver of transgenic medaka line 52 and Wt siblings at 1 year old. Lane 1–5: Wt sibling for line 52, Lane 6–11: HHM transgenic line 52. (PDF) [file pone.0129950.s003.pdf]

**HHM transgenic  
line52**

**Wt siblings for  
line 52**

**HHM transgenic  
line31**

**Wt siblings for line31**

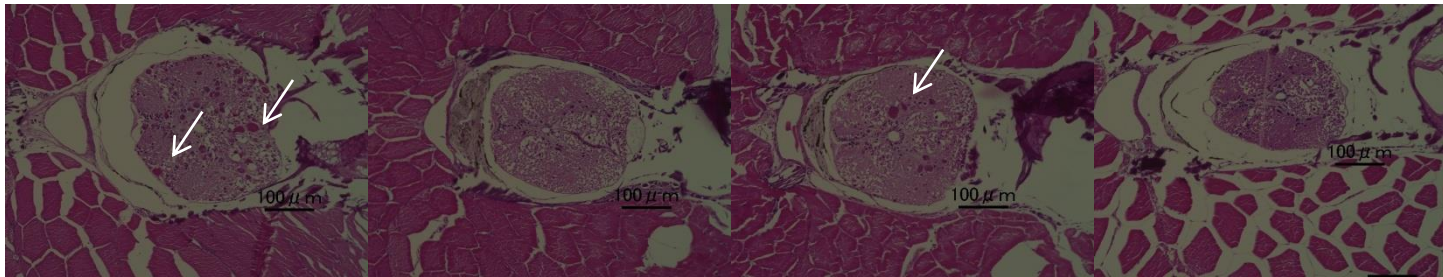

Supplement: S4 Fig — HE staining of cross-sections of spinal cords prepared from the indicated lines of HHM transgenic medaka. Red arrows indicate granular deposits observed only in individuals expressing HHM. (PDF) [file pone.0129950.s004.pdf]

CMV :Maid -GFP

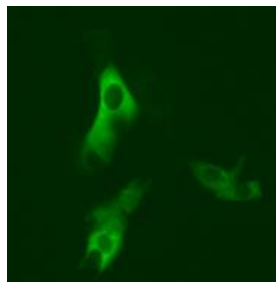

CMV:GFP

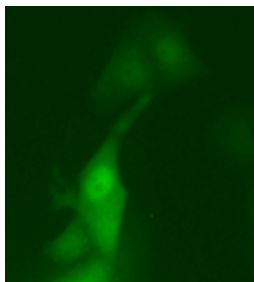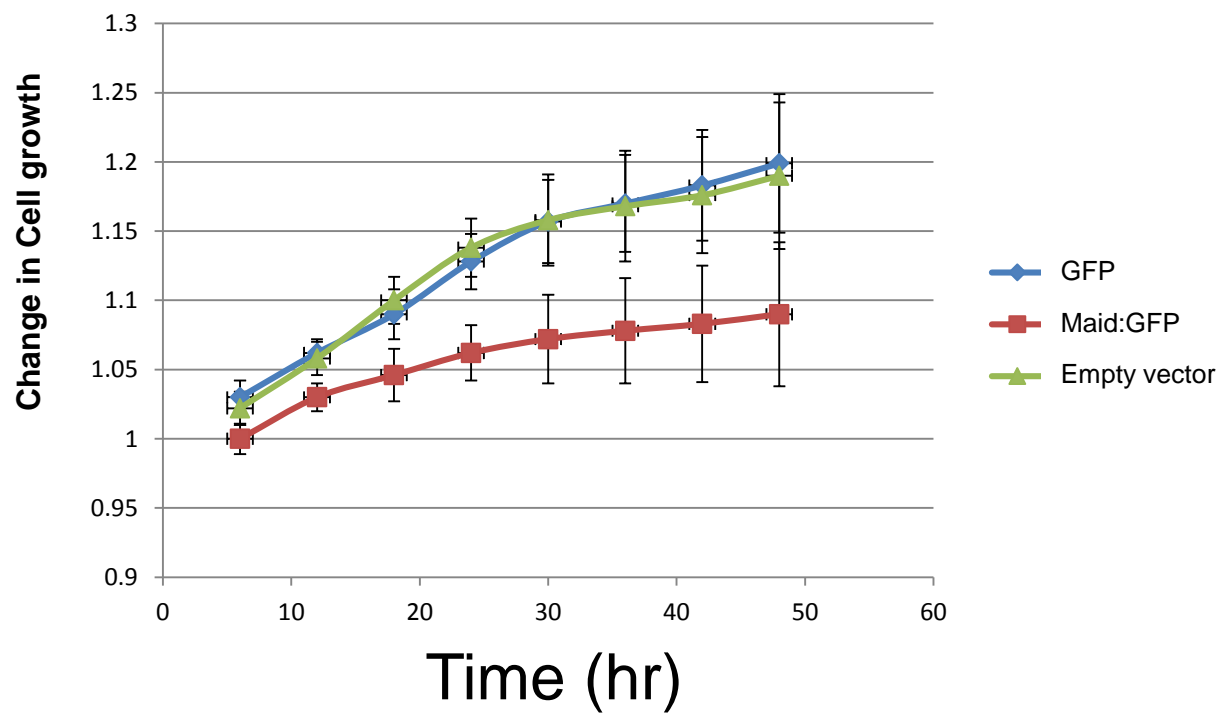

Supplement: S5 Fig — A: Immunofluorescence analysis of (left) ZFL cells expressing Maid-GFP and (right) control ZFL cells expressing GFP. B: Growth curve of the cells. (PDF) [file pone.0129950.s005.pdf]

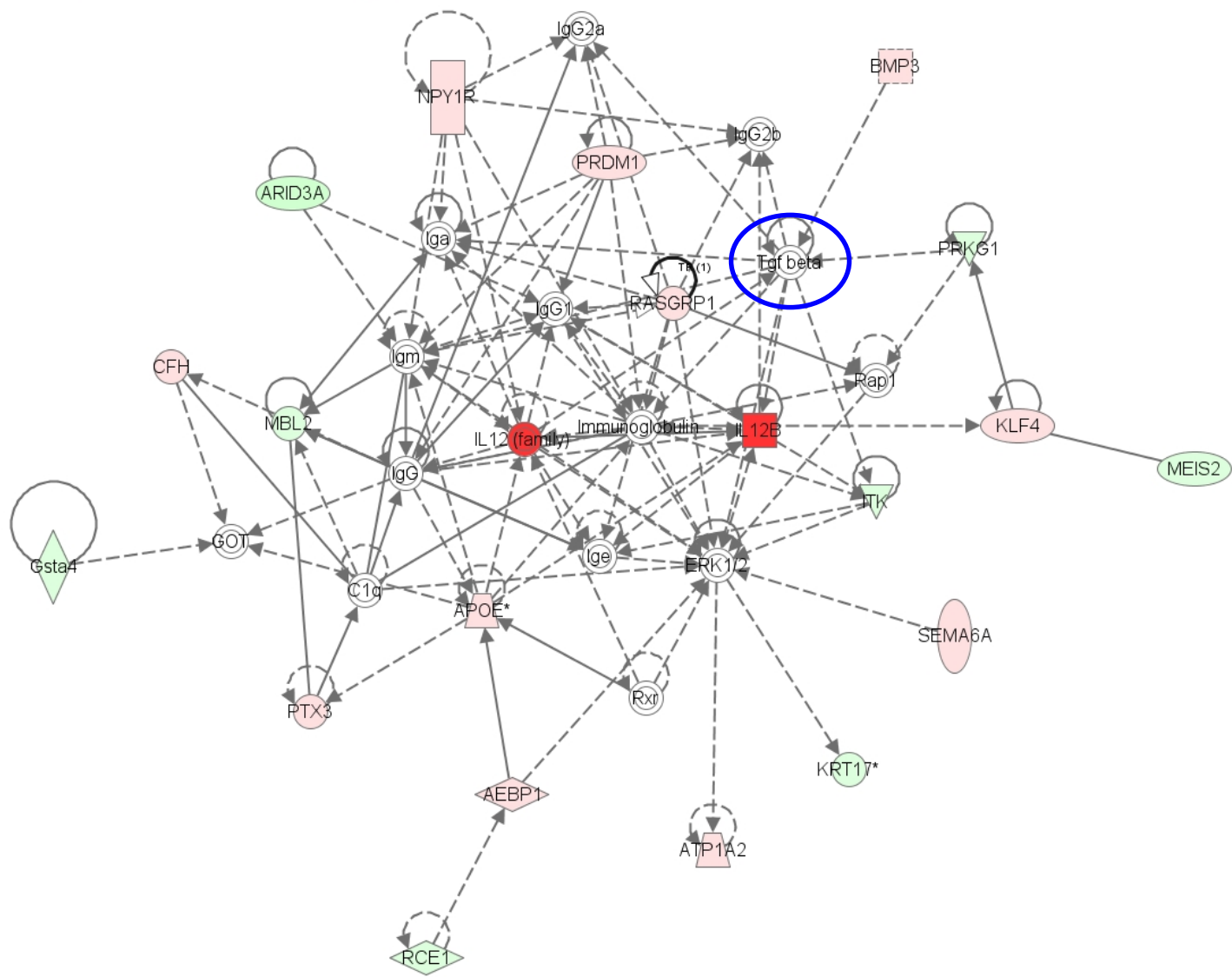

Supplement: S6 Fig — Knowledge-based interaction network of TGF-beta targets after Maid overexpression in ZFL. The network was built based on the TGF-beta interactome in the Ingenuity IPA database overlaid with microarray data from ZFL overexpressing Maid 1.5-fold change cut-off. The intensity of the color indicates the degree of up- (red) or down- (green) regulation. TGF-beta is surrounded by blue line. (PDF) [file pone.0129950.s006.pdf]
