# Supplementary material for: Evidence for a Role of the Transcriptional Regulator Maid in Tumorigenesis and Aging
Source: PLoS One. 2015 Jun 24;10(6):e0129950. doi: 10.1371/journal.pone.0129950 (PMC4479567; doi:10.1371/journal.pone.0129950)
Supplement: S1 Fig — Because initial BLAST data failed to identify Maid homologs in zebrafish, consensus sequences were derived from human and mouse Maid sequences to prepare primers. mRNA was isolated from zebrafish ovaries, which were expected to have the highest expression of Maid mRNA, and cDNA was synthesized to obtain a full-length zebrafish homologue of Maid (ZHM; GenBank, EF581817). The full-length cDNA contained 1210 bp encoding a 344 amino acid protein. (A) Comparison of amino acid sequences of Maid isolated from zebrafish and the indicated species. (*), identical amino acids; (:), highly conserved amino acids; (.), conserved amino acids as determined by Clustal Consensus analysis. HLH, helix-loop-helix motif; LZ, leucine zipper motif. (PDF) [file pone.0129950.s001.pdf]

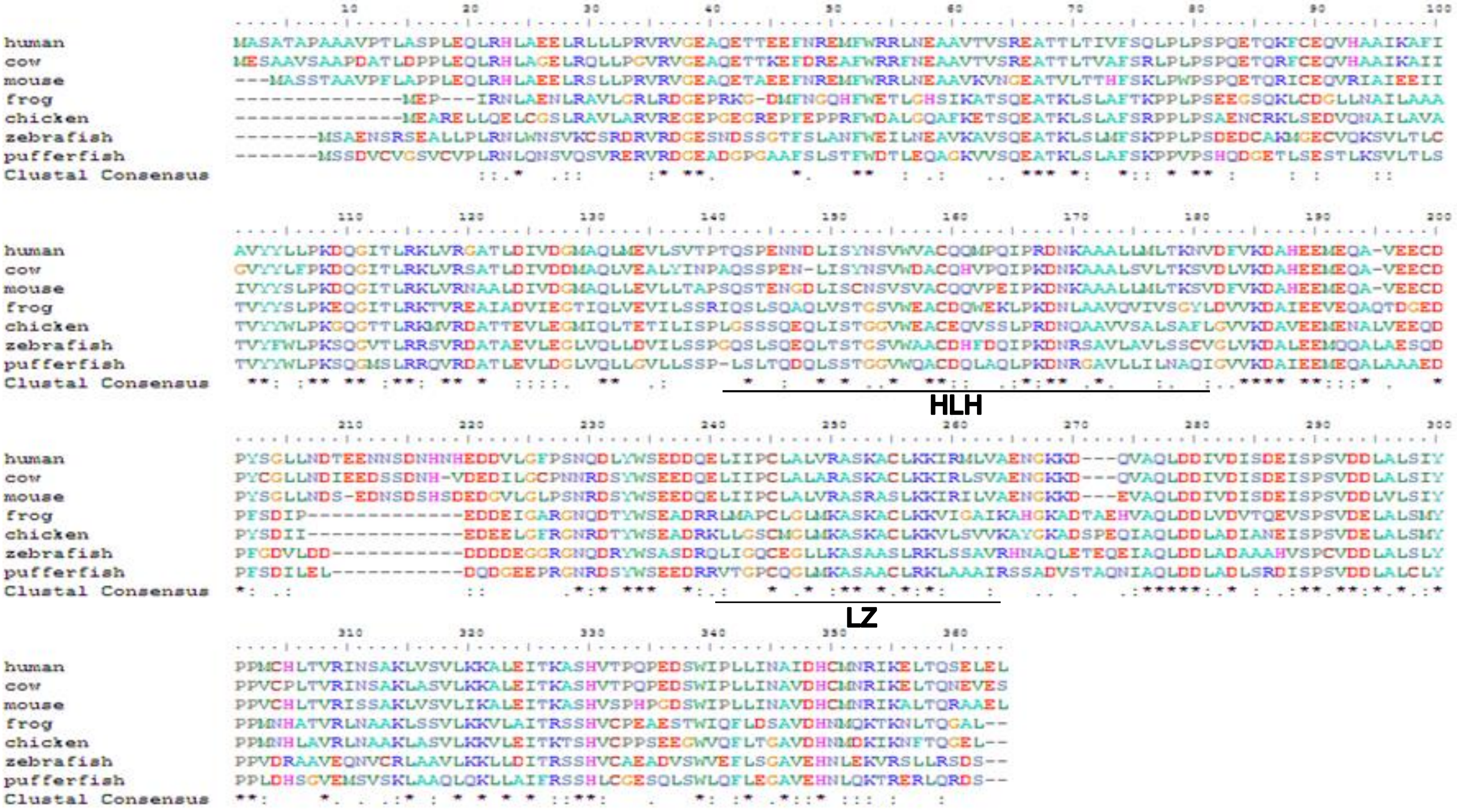
human
cow
mouse
frog
chicken
zebrafish
pufferfish
Clustal Consensus
HLH
LZ
